# Mapping Biodiversity Through Time and Space: Patterns and Drivers of Fabaceae Collection in Mozambique

**DOI:** 10.1002/ece3.72854

**Published:** 2026-02-17

**Authors:** Miguel Brilhante, Iain Darbyshire, Maria Cristina Duarte, Margarida Moldão, Salomão Bandeira, Maria M. Romeiras

**Affiliations:** ^1^ Linking Landscape, Environment, Agriculture and Food (LEAF), Instituto Superior de Agronomia (ISA) Universidade de Lisboa Lisboa Portugal; ^2^ Royal Botanic Gardens, Kew Richmond UK; ^3^ Centre for Ecology, Evolution and Environmental Changes (CE3C) and Change–Global Change and Sustainability Institute, Faculdade de Ciências Universidade de Lisboa Lisboa Portugal; ^4^ Associate Laboratory TERRA, Instituto Superior de Agronomia (ISA) Universidade de Lisboa Lisboa Portugal; ^5^ Department of Biological Sciences Eduardo Mondlane University Maputo Mozambique

**Keywords:** botanical exploration, collection bias, East African flora, herbarium records, Leguminosae, species discovery, type specimens

## Abstract

Despite the extensive diversity of African flora, significant gaps remain in taxonomic research and biodiversity conservation, including under‐sampling in highly diverse regions, a shortage of taxonomic expertise, limited financial resources and delays in species descriptions. Type specimens act as effective proxies for tracking the discovery and description of species, providing a historical baseline for assessing taxonomic effort and our understanding of biodiversity. This study presents the first comprehensive analysis of Fabaceae species collected in Mozambique, one of the most diverse and ecologically important plant families in the region. It offers new insights into the taxonomic, spatial and temporal patterns shaping current botanical knowledge through an analysis of Fabaceae type specimens collected in Mozambique. We identified 273 type specimens, including 126 recognised taxa, with a notable proportion of endemism (44 strict‐endemic and 18 near‐endemic taxa) and a predominance of woody growth forms. Nearly 40% of these taxa lack IUCN conservation assessments, highlighting significant information gaps. The findings reveal that collection activity peaked during colonial botanical initiatives, driven by a small group of prolific collectors and influenced by spatial biases towards southern and central provinces. Using generalised linear modelling, we demonstrate that collection locations were significantly affected by elevation, slope, land cover and proximity to roads and harbours, reflecting the interaction between biogeographic patterns and accessibility. By identifying these historical and geographic biases, our study deepens understanding of Mozambique's botanical heritage and provides a crucial baseline for future floristic and conservation efforts in underexplored regions. Furthermore, this research underscores the vital role of herbarium type specimens as scientific resources supporting taxonomic research and conservation planning, emphasising the importance of preserving and digitising these collections to enhance their accessibility and utility.

## Graphical abstract

Africa's plant diversity remains under‐documented due to limited sampling, expertise and resources, with type specimens offering a historical baseline to evaluate taxonomic effort and biodiversity knowledge. Using generalised linear modelling, we show that collection patterns in Mozambique were shaped by elevation, slope, land cover and accessibility, revealing strong biogeographic and historical biases. These findings highlight major taxonomic and conservation gaps, providing a critical baseline for future floristic and conservation research in underexplored African regions.

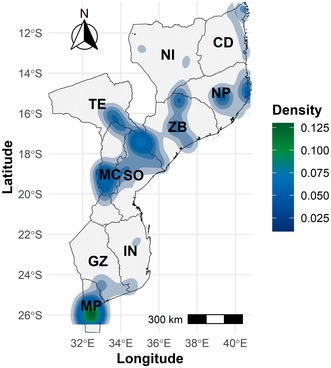

## 1 Introduction

Natural history collections, particularly herbarium specimens, are an invaluable data source for understanding the historical, spatial and social dynamics of plant diversity (Meineke et al. 2019). These data also provide a baseline for detecting shifts and predicting future patterns in biodiversity (Krishtalka and Humphrey 2000). Recent advances in digitisation allow detailed analysis of these data, revealing botanical exploration patterns, taxonomic knowledge gaps and factors influencing biodiversity documentation (Davis 2023; Batke et al. 2025). These data help to identify underexplored regions and biases in botanical sampling effort (Daru and Rodriguez 2023).

Understanding the dynamics of plant diversity over time and space is increasingly urgent in light of accelerating biodiversity loss and the need for informed conservation planning (Meyer et al. 2015). Herbarium data is critical to global initiatives such as the Global Biodiversity Information Facility (GBIF), providing baseline data for biodiversity monitoring, ecological modelling and species threat assessments (Heberling 2022; Eckert et al. 2024).

Among these resources, type specimens stand out due to their essential role in taxonomy, as they are linked to the first formal description of species and infraspecific taxa (Daston 2004; Funk et al. 2005; Proćków et al. 2020). According to the International Code of Nomenclature for algae, fungi and plants, each scientific name is associated with a type specimen, which serves as a definitive taxonomic reference for a given taxon (Turland et al. 2018). Since type specimens anchor the application of scientific names to physical specimens, they are also fundamental for resolving nomenclatural ambiguities and informing conservation assessments, particularly for rare or endemic taxa (Knapp et al. 2004). These type specimens ensure consistency in nomenclature and provide a reliable source for taxonomic research (Swain and Chakraborty 2024).

Herbarium specimens are particularly valuable for studying the floristic composition of poorly documented regions, such as Mozambique, where significant knowledge gaps persist (Romeiras et al. 2014; Darbyshire et al. 2019). Despite the increasing availability of digitised and georeferenced data from African herbaria (Sosef et al. 2017), such data still reflect considerable geographical, temporal and taxonomic biases (Meyer et al. 2015; Daru et al. 2018), which may affect the interpretation of biodiversity patterns. Nonetheless, recognising and quantifying these biases is crucial for guiding future collection efforts and conservation priorities.

With over 6000 currently recognised plant taxa (Odorico et al. 2022), including 271 strict endemics and 387 near endemics, Mozambique is a major centre of plant biodiversity in Africa (Darbyshire et al. 2019, 2023). This remarkable diversity results from a combination of tropical and subtropical climates along with a variety of habitats, such as Afromontane forests, inland savannas and coastal plains (e.g., Bandeira et al. 1996). Among the most species‐rich families is Fabaceae (*nom. alt*. Leguminosae), which encompasses a wide range of growth forms, including annual and perennial herbs, climbers, shrubs and trees, inhabiting nearly all habitats and phytogeographic regions of the country (Lewis et al. 2005; Hyde et al. 2025). This family is ecologically dominant across tropical and subtropical biomes and has been the focus of major global phylogenetic efforts, such as those led by the Legume Phylogeny Working Group (LPWG) (2017). Beyond their taxonomic diversity, species of Fabaceae are functionally versatile, being used for food, fodder, medicine, construction, timber, ornamentals and even in social rituals within local communities (e.g., Ribeiro et al. 2010; Sitoe and Van Wyk 2024). In addition to their ecological and economic importance, Fabaceae species in Mozambique include several wild relatives of domesticated crops, with the genus *Vigna* standing out, positioning the country as a globally significant reservoir of Crop Wild Relatives (CWRs) (Brilhante et al. 2023). Due to their representativeness, broad distribution, ecological importance and high levels of endemism, Fabaceae constitutes a highly suitable group for studying patterns of diversity and conservation within Mozambique's flora (Darbyshire et al. 2019).

However, despite the importance of Fabaceae as a diverse and ecologically significant plant family, the historical and spatial processes underlying their documentation in Mozambique have not been thoroughly analysed. Understanding how species have been collected and described over time offers insights into biodiversity patterns and the sociopolitical and logistical factors that shaped botanical exploration in the country. Therefore, by examining type specimens, we aim to evaluate how Fabaceae biodiversity in Mozambique has been explored, collected and formally described over time, as well as the factors that have influenced these patterns. To this end, we pursued the following specific objectives: (i) to assess the diversity and conservation of type specimens, including their native status, growth form and IUCN conservation status, to evaluate the representativeness and conservation relevance; (ii) to identify the most prolific collectors of type specimens in Mozambique; (iii) to analyse temporal trends in the collection and formal description of species, including the rate of species discovery and the time lag between collection and publication; (iv) to investigate spatial patterns of type specimen collection, identifying geographical hotspots of collection effort and (v) to assess the influence of environmental factors (i.e., elevation, slope and land cover/land use) and anthropogenic factors (i.e., distance to roads and harbours) on the presence and distribution of type specimen collections using statistical models. Through our approach, we also aim to provide guidelines for future taxonomic and conservation efforts by focusing on regions underrepresented in type specimen records. Although these records are specific to Fabaceae types, they may indicate broader gaps in plant sampling and help identify potential priorities for targeted botanical exploration in Mozambique.

## 2 Methodology

### 2.1 Studied Area

Mozambique is in the southeast of the African continent, between latitudes 10°20′ S and 26°50′ S, covering an area of approximately 801,590 km^2^ (Figure S1). The country shares borders with Tanzania, Malawi, Zambia, Zimbabwe, Eswatini, South Africa and the Indian Ocean, featuring a coastline of over 2800 km (Palalane et al. 2016). It is divided into 11 provinces (i.e., Cabo Delgado, Niassa, Nampula, Zambezia, Tete, Manica, Sofala, Gaza, Inhambane, Maputo and Maputo City), with Maputo being its capital.

The country boasts a diverse range of ecosystems, shaped by its geographical and topographical complexity, including lowland and montane forests, miombo and mopane woodlands, coastal mosaics, wetlands and mangroves (Bandeira et al. 1996; Joaquim‐Meque et al. 2023). According to the national historical vegetation and Red List of Ecosystems assessment, Mozambique hosts 162 recognised ecosystem types, reflecting its wide ecological heterogeneity (Lötter et al. 2023). Forests and woody vegetation dominate the land cover, accounting for approximately 70% of the national territory, with around 26% designated as protected areas (MICOA 2014). This diversity supports a high level of plant richness (Odorico et al. 2022) and several centres of endemism, such as Chimanimani‐Nyanga, Rovuma and Maputaland (Darbyshire et al. 2019), as well as the South East Africa Montane Archipelago (Bayliss et al. 2024).

Mozambique's climate is predominantly tropical, with subtropical conditions in the southern inland (Barbosa et al. 2001). The country has a hot, humid season from November to March and a cooler, dry season from April to October (Hoguane 2007). Average annual rainfall varies greatly, from around 300 mm in the south to over 1400 mm in the Zambezi valley (Uamusse et al. 2017).

### 2.2 Data Collection

#### 2.2.1 List of Fabaceae Type Specimens

A comprehensive dataset was compiled primarily by analysing Fabaceae specimens collected in Mozambique and available online through GBIF.org (2025) and JSTOR Global Plants (2025). For each specimen, all information was extracted manually through direct examination of high‐resolution specimen scans and associated metadata, including details from specimen labels (e.g., collector, catalogue number, collection number, locality, collection date and type status) and digital record fields (e.g., ‘Record number’, ‘Recorded by’, ‘Institution code’, ‘Catalogue number’, ‘Event date’ and ‘Locality’) to ensure accurate attribution (Table S1). These specimens are deposited in the following herbaria: Herbarium of the Natural History Museum (BM), Herbarium of the University of Bologna (BOLO), Herbarium of the Meise Botanical Garden (BR), Herbarium of the University of Coimbra (COI), Selmar Schonland Herbarium of the Albany Museum, South Africa (GRA), Herbarium of the University of Hamburg (HBG), Herbarium of the University of Oxford (FHO), Herbarium of the Royal Botanic Gardens, Kew (K), Herbarium of the Institute of Tropical Scientific Research, University of Lisbon (LISC), Herbarium of Missouri Botanical Garden (MO), Herbarium of the Institute for Agricultural Research of Mozambique (LMA), Herbarium of Eduardo Mondlane University (LMU), Bews Herbarium of the University of KwaZulu‐Natal (NU), Herbarium of The New York Botanical Garden (NY), Herbarium of the Muséum National d'Histoire Naturelle, Paris (P), Herbarium of the University of Porto (PO), Herbarium of the South African National Biodiversity Institute (PRE), Herbarium of the Naturalis Biodiversity Center (WAG), Herbarium of the University Vienna (WU) and Herbarium of the University of Zurich (Z).

The data extracted from herbarium specimens were supplemented with information obtained from key bibliographic sources focused on African flora, including Flora of Tropical Africa (Baker 1871, 1926, 1929, 1930), Flora of Tropical East Africa (Brenan 1967; Verdcourt 1970a, 1970b, 1970c, 1970d, 1971; Gillett et al. 1971a, 1971b) and Flora Zambesiaca (Brenan 1970; Schrire 1998, 2012; Verdcourt 2000; Mackinder et al. 2001; Pope et al. 2003; Brummitt et al. 2007a, 2007b). These sources were used to supplement and verify specimen‐based data, providing additional information on taxon names and synonyms, protologue references, collector names, collection numbers, collection localities and publication details. Additional relevant online databases were also consulted, namely the African Plant Database (APD 2025), Flora of Mozambique (Hyde et al. 2025), IUCN Red List of Threatened Species (IUCN 2025), International Plant Names Index (IPNI 2025), Plants of the World Online (POWO 2025) and Tropicos (Tropicos 2025).

A detailed dataset of Fabaceae type specimens collected in Mozambique was compiled. It includes the following information for each specimen (when available): scientific name (and synonym(s), when applicable), native status, growth form, IUCN extinction risk (conservation) status, native distribution, protologue, collector name, collection number, collection locality, collection year, herbarium institution(s) and the unique herbarium identifier (barcode or accession number) (see Table S1).

#### 2.2.2 Type Specimen Occurrence Data

The geographic data of the type specimens were obtained through georeferencing of the locality descriptions provided on the herbarium specimen labels. This process followed the guidelines of Zermoglio et al. (2020) and was carried out using Google Earth Pro 7.3.4.8573 (Serea 2018) and QGIS v.3.4.15. (QGIS Development Team 2025). Although a total of 273 Fabaceae type specimens collected in Mozambique were identified, not all could be georeferenced due to insufficient or vague locality information. As a result, the final dataset comprised 258 records of Fabaceae type specimens, which served as the basis for all the spatial analyses conducted in this study (Table S1).

### 2.3 Collector Analysis

The most prolific collectors were identified, and their contributions were summarised to assess their influence on the discovery of new Fabaceae species in Mozambique. The collector names were standardised and disaggregated into individual contributors, accounting for joint collections frequently recorded under combined names. For each collector, we calculated three metrics adapted from Bebber et al. (2012): total number of type specimens ($n_{\mathrm{esp}}$), number of distinct provinces where collections were made ($n_{\text{locality}}$) and duration of collecting activity (difference between earliest and latest collection year; duration). Additionally, records lacking a collection year or location were flagged to quantify data incompleteness. All metrics were rescaled to a 0–1 range using min‐max normalisation and integrated into an index of collector importance using Formula (1):

(1) $${\text{Index}}_{\text{collector}}=0.5\times n_{\mathrm{esp}}+0.3\times n_{\text{locality}}+0.2\times \text{duration}$$

This weighting scheme, proposed here for the first time, reflects the assumed relative contribution of each metric to collector importance, giving greater influence on the number of specimens collected (50%), followed by spatial (30%) and temporal (20%) coverages. While alternative schemes are possible, the analysis indicated that the ranking of major collectors remained largely stable under moderate variations in these values, supporting the robustness of this approach.

The ten collectors with the highest index values were selected for further analysis (Table S2). Their collection efforts were then examined through heatmaps, showing specimen numbers per decade and province. Records with missing collection years were manually assigned to plausible time breaks (e.g., collections by Wilhelm Peters were grouped under the 1840s based on historical expedition records; Liberato 1994). Provinces with unknown data were omitted from the spatial analysis. A Spearman correlation test was used to assess the relationship between collection duration and the number of type specimens collected.

### 2.4 Temporal and Spatial Trends

To characterise the temporal dynamics of Fabaceae type specimen collection in Mozambique, specimen records were aggregated by both the year of collection and the year of formal publication of the taxon description (i.e., protologue). Following the approach of Romeiras et al. (2020), discovery accumulation curves were generated to observe the progression of botanical knowledge over time. The time lag between collection and publication was calculated for each taxon as the difference between the earliest specimen collection date and the publication year of the protologue. Time‐lag values were then grouped and averaged by the decade of the collection year, which best reflects the period in which specimens first entered scientific knowledge. Temporal trends were visualised with bar and line plots, including dual y‐axes to show both absolute values and cumulative proportions. Neotypes were documented in Table S1 for completeness; however, they were excluded from all temporal analyses. Because neotypes are designated long after the original protologue, their collection dates do not reflect the historical context of taxon discovery and would therefore generate misleading or biologically meaningless time‐lag values. For this reason, only holotypes, isotypes, syntypes, isosyntypes, paratypes, isoparatypes and lectotypes were retained in the temporal analysis. However, when multiple specimens were recorded for the same taxon (namely, syntypes and paratypes), only the earliest specimen was considered, thereby avoiding pseudoreplication while capturing the historical pattern of taxon discovery.

Spatial trends analysis was conducted in R v.4.3.3 (R Core Team 2025), primarily using the packages ‘sf’ (Pebesma 2018), ‘ggplot2’ (Wickham 2016), ‘ggspatial’ (Dunnington 2023) and ‘dplyr’ (Wickham et al. 2023). Specimen coordinates were mapped and coloured by collection decade using ‘geom_sf’. Spatial collection density was estimated using Kernel Density Estimation (KDE) through ‘stat_density_2d’, allowing the identification of hotspots of collection activity and potential undersampled regions. Map features were enhanced with ‘annotation_north_arrow’ and ‘annotation_scale’ from the ggspatial package.

Specimen records were also analysed against ecologically meaningful spatial layers to better capture patterns relevant to biodiversity and conservation, complementing the province‐level analysis. These included: (i) terrestrial ecoregions (Dinerstein et al. 2017) occurring within Mozambique, namely Central Zambezian wet miombo woodlands, Dry miombo woodlands, Limpopo lowveld, Maputaland coastal forests and woodlands, Mulanje Montane forest grassland, Nyanga‐Chimanimani Montane forest‐grassland, Southern Africa mangroves, Southern Rift Montane forest‐grassland, Southern Swahili coastal forest and woodlands, Zambezian coastal flooded savanna, Zambezian flooded grasslands, Zambezian Mopane woodlands and Zambezian‐Limpopo mixed woodland; Centres of Endemism (CoE, Darbyshire et al. 2019), namely Rovuma, Maputaland, Lebombo Mountains, Inhambane and Chimanimani‐Nyanga, together with the recently described South East African Montane Archipelago (Bayliss et al. 2024); and Important Plant Areas (IPAs; Darbyshire et al. 2023).

### 2.5 Statistical Modelling of Spatial Patterns

#### 2.5.1 Predictor Variables

A set of environmental accessibility‐related predictor variables was considered to investigate the environmental and anthropogenic factors influencing the collection patterns of Fabaceae type specimens in Mozambique. All spatial data were processed in R using the ‘sf’ (Pebesma 2018), ‘terra’ (Hijmans 2025), ‘raster’ (Hijmans 2025), ‘extactextractr’ (Baston 2023) and ‘dplyr’ (Wickham et al. 2023) packages. Layers were standardised to the WGS84 coordinate reference system using the nearest neighbour resampling method.

The environmental predictors included elevation, slope and land use/land cover (LULC). Elevation data were sourced from WorldClim v2.1 (WorldClim 2020) at a spatial resolution of 2.5 arc minutes (ca. 5 km) (Fick and Hijmans 2017). Slope was derived in degrees from the elevation raster by using the ‘terrain ()’ function of the ‘terra’ package. LULC data were obtained from Copernicus Climate Change Service (2019) and reclassified into six relevant ecological categories as follows: (1) Agriculture and Croplands, (2) Forest and Tree Cover, (3) Grassland and Shrubland, (4) Wetland and Riparian Zones, (5) Urban and Bare Areas and (6) Water Bodies. All raster layers were loaded and aligned to the same spatial projection, and the values corresponding to each specimen record were extracted using the ‘extract()’ function from the ‘terra’ package.

Anthropogenic variables related to accessibility were also considered, including the distances to the nearest road and harbour. Shapefiles including the roads were sourced from Humanitarian Data Exchange (2024) and harbour locations from the National Geospatial‐Intelligence Agency (NGA) (2025), respectively. These distances were previously calculated in QGIS v.3.4.15 (QGIS Development Team 2025) using the ‘Distance to Nearest Hub’ function and stored as shapefile attributes. After being imported into the R environment as ‘sf’ objects, the distance values were associated with each specimen record using the ‘st_nearest_feature’ function of the ‘sf’ package. Because the LULC and accessibility layers represent current conditions, collection site descriptions from specimen labels were examined when available to reduce potential temporal inconsistencies. While the exclusion of imprecisely georeferenced specimens greatly reduced positional uncertainty, some residual inaccuracy may remain, particularly for historical records; nevertheless, the resulting estimates are adequate for identifying broad‐scale spatial patterns of collection.

To enable binary analysis using logistic models, pseudo‐absences were generated within the geographical extent of the presence data. The ‘st_sample’ function was applied to randomly generate a set of points within the bounding box of the recorded specimens, maintaining a balanced proportion between presences and absences. Environmental and accessibility variables were extracted from each pseudo‐absence, following the same procedure applied to the presence data.

A final data frame was built, including the columns corresponding to the binary response variables (presence = 1 and pseudo‐absence = 0) and all predictor variables. The LULC variable, being categorical, was converted into factors with standardised levels.

Before model fitting, an exploratory analysis of the predictor variables was conducted using histograms and bar plots to visually assess their relationship with type specimen collection occurrences, as presented in Figure S2.

#### 2.5.2 Generalised Linear Model

To model the spatial pattern of Fabaceae type specimen collection in Mozambique, we applied a generalised linear model (GLM) with binomial distribution and logit link function, appropriate for the analysis of presence/absence data (McCullagh and Nelder 1989). The binary response variable indicated the presence (1) or pseudo‐absence (0) of type specimen collection records. The predictor variables included combinations of environmental factors (i.e., elevation, slope and LULC) and anthropogenic variables related to accessibility (i.e., distance to the nearest road and harbour). Therefore, the general model applied is described by Equation (2):

(2) $$\text{logit}\left(P\right)=\beta_{0}+\beta_{1}X_{1}+\beta_{2}X_{2}+\ldots +\beta_{n}X_{n}$$

where P is the probability of a type specimen being collected in a given location, $\beta_{0}$ is the intercept and $\beta_{1}\ldots \beta_{n}$ are the estimated coefficients associated with each predictor variable $X_{1}\ldots X_{n}$. The tested models included different combinations of these predictors to evaluate their influence on collection patterns.

A full model was initially fitted with all the variables. Subsequently, reduced models with specific combinations of predictor variables and a null model (intercept only) were tested to compare different hypotheses. The selection of the best model was based on the corrected Akaike Information Criterion (AICc), using the ‘AICc()’ function of the AICcmodavg package (Mazerolle 2023). To assess the quality of fit, McFadden's pseudo‐*R* ^2^ was calculated manually, following McFadden (1974). To evaluate the predictive performance, the area under the curve (AUC) of the receiver operating characteristic (ROC) was computed using the ‘pROC’ package (Robin et al. 2011). The several models were then compared using the AICc, and the model with the lowest AICc and highest Pseudo‐*R* ^2^ was selected as the best fit, as described by Roxo et al. (2021).

## 3 Results

### 3.1 Diversity of Fabaceae Type Specimens

A total of 273 Fabaceae type specimens collected in Mozambique were identified, of which 178 (65%) are types of currently accepted names, representing 126 distinct taxa, with some taxa having more than one type specimen (e.g., syntypes or paratypes; see Table S1). The remaining 95 (35%) type specimens correspond to scientific names currently treated as taxonomic synonyms. Only type specimens associated with accepted taxa were considered in the diversity analysis, since synonymised names do not represent distinct biological entities and therefore cannot be reliably assigned morphological or conservation attributes. Nevertheless, these synonymous type specimens retain taxonomic and historical significance and are subsequently analysed.

Table 1 summarises the distribution of the 126 accepted taxa according to native status, growth‐form and IUCN categories. The most represented genera were *Indigofera* (**21**), *Crotalaria* (**15**) and *Tephrosia* (**13**), together accounting for approximately 40% of the specimens. Regarding the native status, 64 (51%) taxa are native non‐endemics, 44 (35%) are strict‐endemics and 18 (14%) are near‐endemics. Additionally, seven of the endemic taxa are known only from their type specimen. In terms of growth form, woody plants (trees and shrubs) dominate overall, although the three most represented genera are mainly herbaceous or subshrubs. Specifically, shrubs or subshrubs account for 44 taxa (35%), followed by 27 (21%) annual or biennial herbs, 26 (21%) perennial herbs, 25 (20%) trees, 3 (2%) woody climbers and 1 (1%) perennial climbing herbs. Of the 126 taxa, only 80 (63%) have been evaluated on the IUCN Red List of Threatened Species, while 46 (37%) remain unevaluated. Among the evaluated taxa, 46 taxa (37%) are classified as Least Concern (LC), 14 (11%) as Vulnerable (VU), 6 (5%) as Endangered (EN), 4 (3%) as Critically Endangered (CR) and nine (7%) as Data Deficient (DD).

**TABLE 1:** Distribution of taxa by growth form, native status and IUCN conservation status among the 126 accepted Fabaceae type taxa collected in Mozambique.

Character	No. of taxa	% of taxa
Native status		
Near‐endemic	18	14
Native non‐endemic	64	51
Strict‐endemic	44	35
Growth form		
Annual or biennial herb	27	21
Perennial herb	26	21
Perennial climbing herb	1	1
Shrub or subshrub	44	35
Tree	25	20
Woody climber	3	2
IUCN		
Critically endangered (CR)	4	3
Endangered (EN)	6	5
Vulnerable (VU)	14	11
Near threatened (NT)	1	1
Least concern (LC)	46	37
Data deficient (DD)	9	7
Not evaluated (NE)	46	37

*Note:* Percentages refer to the proportion of total accepted taxa.

### 3.2 Temporal Trends of Type Specimen Collection

**FIGURE 1:**
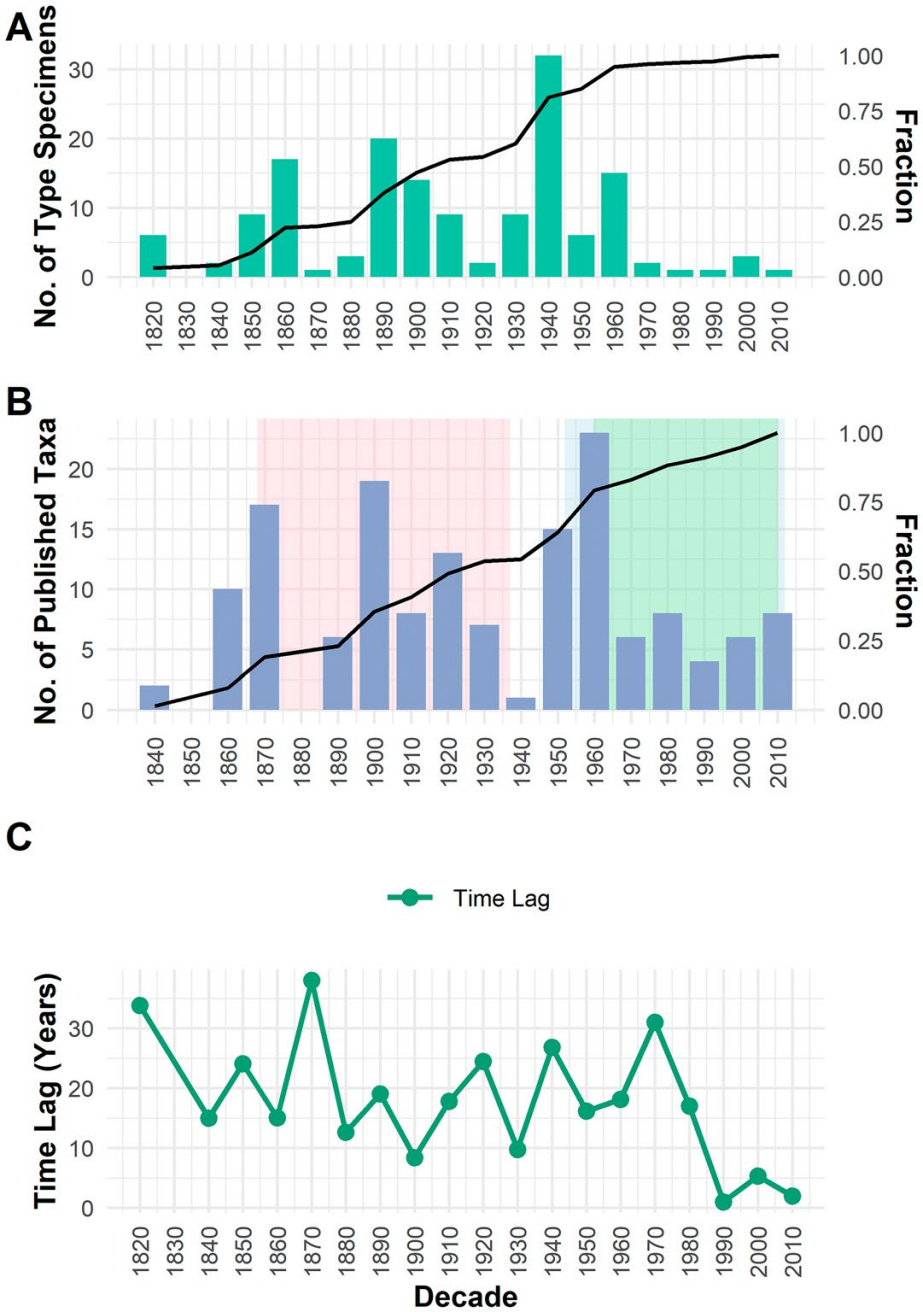
Temporal trends in the collection and publication of type specimens. (A) Number of type specimens recorded per decade (green bars) and their fraction relative to the total specimens collected (black line). (B) Number of published taxa per decade (blue bars) and their fraction relative to the total type specimens (black line). Shaded areas highlight the publication of ‘Flora of Tropical Africa’ (1868–1937), ‘Flora of Tropical East Africa’ (1952–2012) and ‘Flora Zambesiaca’ (1960–present). (C) Time lag between the collection of the first type specimen and the publication of the respective taxon, along with the cumulative number of published taxa. Data prior to 1820 were excluded from the analyses due to data scarcity.

The temporal analysis of the collection of type specimens of Fabaceae in Mozambique revealed distinct phases of collection and formal description of the taxa (Figure 1). Although the first record dates to 1770 (Table 1), data before the 1820s were excluded from the analyses due to data scarcity. From 1820 onwards, specimen collection gradually increased throughout the 19th century and became more pronounced in the 20th century, with collection peaks in the 1860s, 1890s, 1940s and 1960s with 17, 20, 32 and 15 specimens, respectively (Figure 1A). The 1940s, therefore, marked the period with the highest collection intensity. From the 1970s onwards, there was a sharp drop in specimen collection, which only partially recovered from the 2000s onwards. Around 80% of the type specimens of Fabaceae from Mozambique were collected up until 1940. The number of taxa published over time (Figure 1B) follows the trend of the number of specimens collected. Figure 1C illustrates the time difference between the date of collection and the date of publication, showing a dynamic trend over time with an average lag of 18 years. In the early decades (1770–1890), the time between collecting and describing the Fabaceae was long and variable, reaching more than 30 years in 1870, with few taxa described. In the 20th century, this interval decreased, and the number of descriptions increased, especially between 1950 and 1960. In recent decades, the description time has fallen to a few years, while the number of new species described has decreased, indicating stabilisation. The cumulative total of described species increased progressively until it reached 153 taxa in 2010.

### 3.3 Key Collectors of Fabaceae Discovery in Mozambique

Detailed information on collectors, including the number of Fabaceae type specimens collected, collecting activity over the years, and spatial coverage by provinces, is summarised in Table S2. This table also features a composite index of collector importance used to identify the ten most prolific contributors.

A total of 64 collectors were responsible for collecting Fabaceae type specimens, with the ten most prolific collectors accounting for over 70% of all type specimens. The earliest recorded Fabaceae type specimen, *Cordyla africana* Lour., was collected in 1770 by João Loureiro, although its exact location is unknown. The most recent, *Bauhinia burrowsii* E.J.D.Schmidt, was collected by Ernst Schmidt in 2010 in Inhambane province.

**FIGURE 2:**
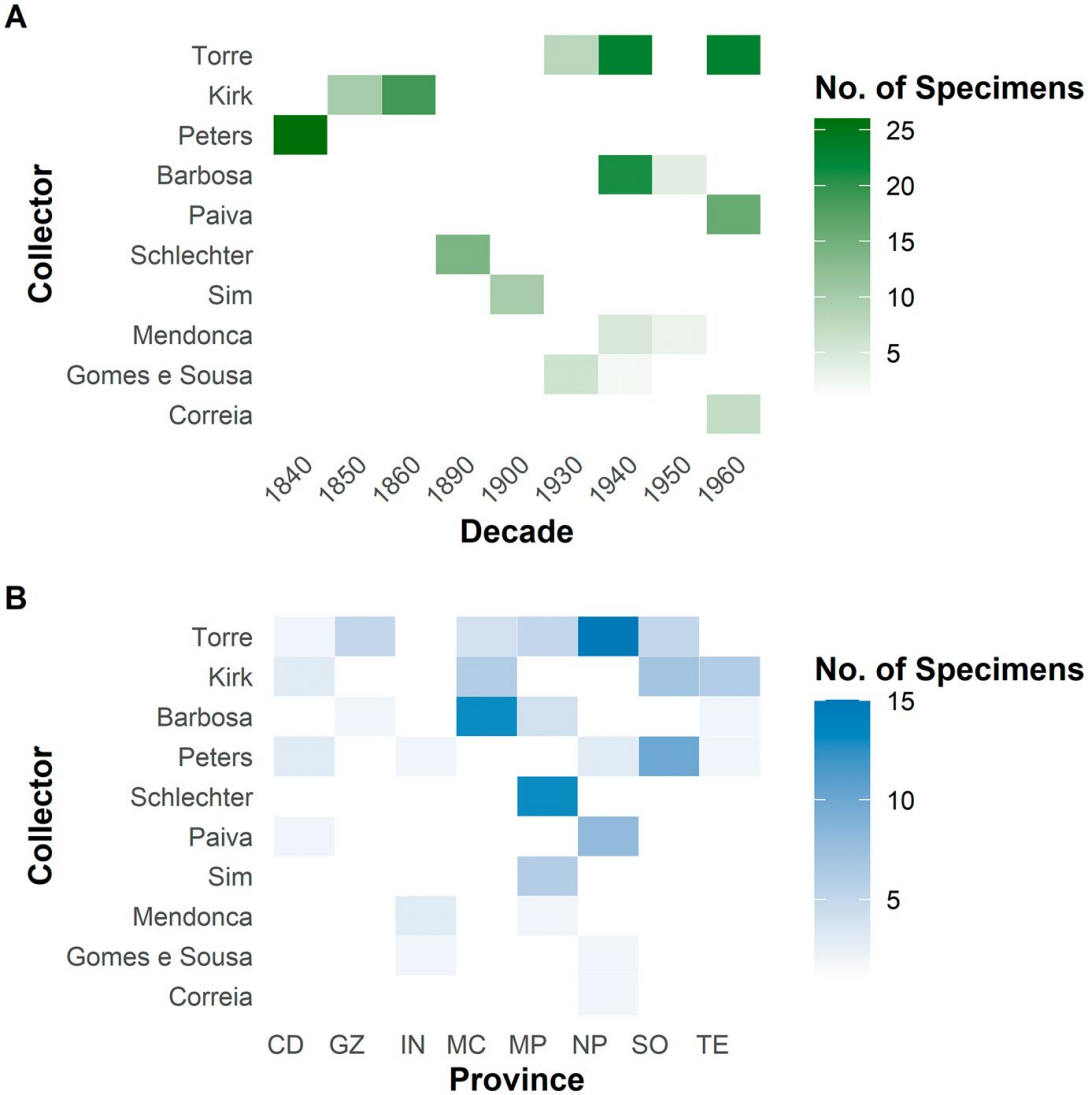
Heatmaps displaying the temporal and spatial distribution of type specimen collections by major collectors in Mozambique. (A) Number of specimens collected per collector across 10‐year intervals. (B) Number of specimens collected per collector across provinces.

**FIGURE 3:**
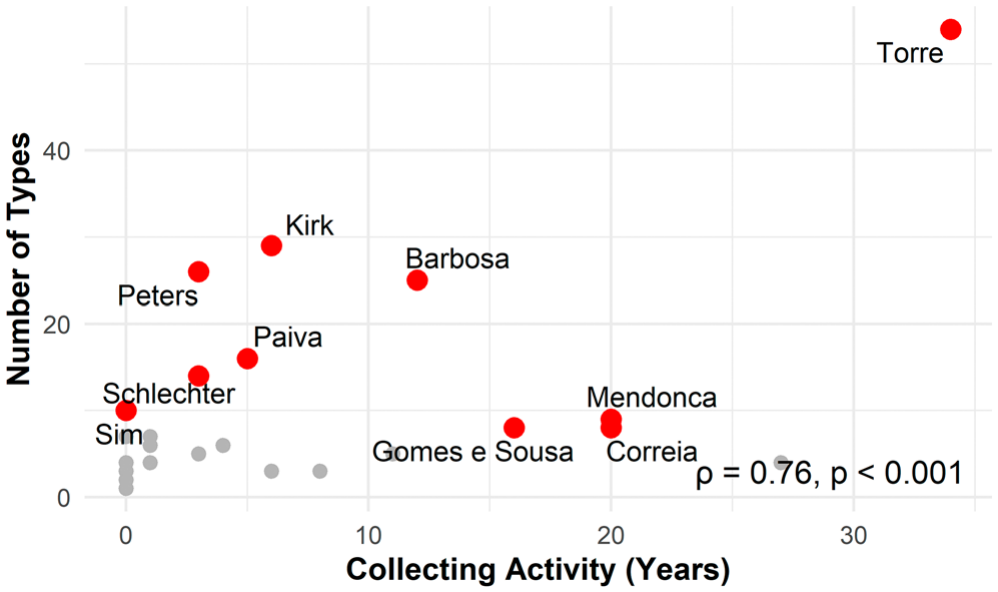
Relationship between the duration of collecting activity and the number of type specimens collected by individual collectors in Mozambique. Each point represents one collector; the top 10 collectors by number of type specimens are highlighted in red and labelled. The dashed line shows the linear regression fit. The Spearman correlation coefficient (*ρ*) and significance (*p* value) are indicated in the figure.

Figure 2 displays the temporal (Figure 2A) and spatial (Figure 2B) patterns of type specimen collection by the major collectors. Among the ten most prolific collectors, António Rocha da Torre stands out as the most productive, having collected 54 type specimens between 1934 and 1968. Although collected widely, a significant proportion of his type specimens of Fabaceae originated from Cabo Delgado, Inhambane, Gaza, Manica, Maputo, Nampula, Niassa, Sofala, Tete and Zambezia provinces. John Kirk and Wilhelm Peters follow, contributing 29 and 26 type specimens, respectively, during the mid‐19th century. Details for other major collectors are provided in Figure 2 and Table S1. Furthermore, the analysis of the relationship between the duration (in years) of each collector's collecting activity and the number of specimens collected revealed a significant positive correlation (Spearman, *ρ* = 0.63, *p* < 0.001), as illustrated in Figure 3.

### 3.4 Factors Shaping Collection Patterns

To assess the factors influencing the collection patterns of Fabaceae type specimens in Mozambique, several spatial and statistical analyses were conducted. The spatial distribution of Fabaceae type collections across the provinces of Mozambique showed that the highest numbers were recorded in Maputo (50 specimens), followed by Sofala (40), Zambezia (39), Manica (34) and Nampula (31), which together accounted for more than 70% of the total specimen collections. Conversely, the provinces of Cabo Delgado (19 specimens), Tete (14), Inhambane (12), Niassa (12) and Gaza (seven) exhibited lower numbers of collected specimens. An analysis across ecologically and conservation‐relevant spatial units revealed distinct distribution patterns of Fabaceae type specimens (Table 2). Important Plant Areas (IPAs) accounted for 13% of the total specimens. Centre of Endemism (CoE) presented the highest representation in Rovuma (19%) and Maputaland (14%), followed by the South East African Montane Archipelago (9%), Inhambane (5%), Lebombo Mountains (4%) and Chimanimani‐Nyanga (4%). Regarding ecoregions, most type specimens were collected within the Southern Swahili coastal forests and woodlands (30%) and Dry miombo woodlands (26%), while other ecoregions, such as the Central Zambezian wet miombo woodlands (1%), contributed fewer records. No records were recorded within Mulanje Montane forest‐grassland, Nyanga‐Chimanimani Montane forest‐grassland, Southern Africa mangroves, Southern Rift Montane forest‐grassland and Zambezian flooded grasslands.

**TABLE 2:** Distribution of Fabaceae type specimens across terrestrial ecoregions, Centres of Endemism (CoE) and important plant areas (IPAs).

Category	No. of specimens	% of specimens
IPAs		
All IPAs combined	33	13
CoE		
Chimanimani‐Nyanga	11	4
Inhambane	14	5
Lebombo Mountains	11	4
Maputaland	37	14
Rovuma	48	19
South East African Montane Archipelago	22	9
Ecoregions		
Central Zambezian Wet Miombo Woodlands	3	1
Dry Miombo Woodlands	67	26
Limpopo Lowveld	9	4
Maputaland Coastal Forests and Woodlands	47	18
Southern Swahili Coastal Forests and Woodlands	78	30
Zambezian Coastal Flooded Savanna	10	4
Zambezian Limpopo Mixed Woodlands	26	10
Zambezian Mopane Woodlands	18	7

*Note:* Percentages represent the proportion of specimens within each category relative to the total dataset (*n* = 258).

**FIGURE 4:**
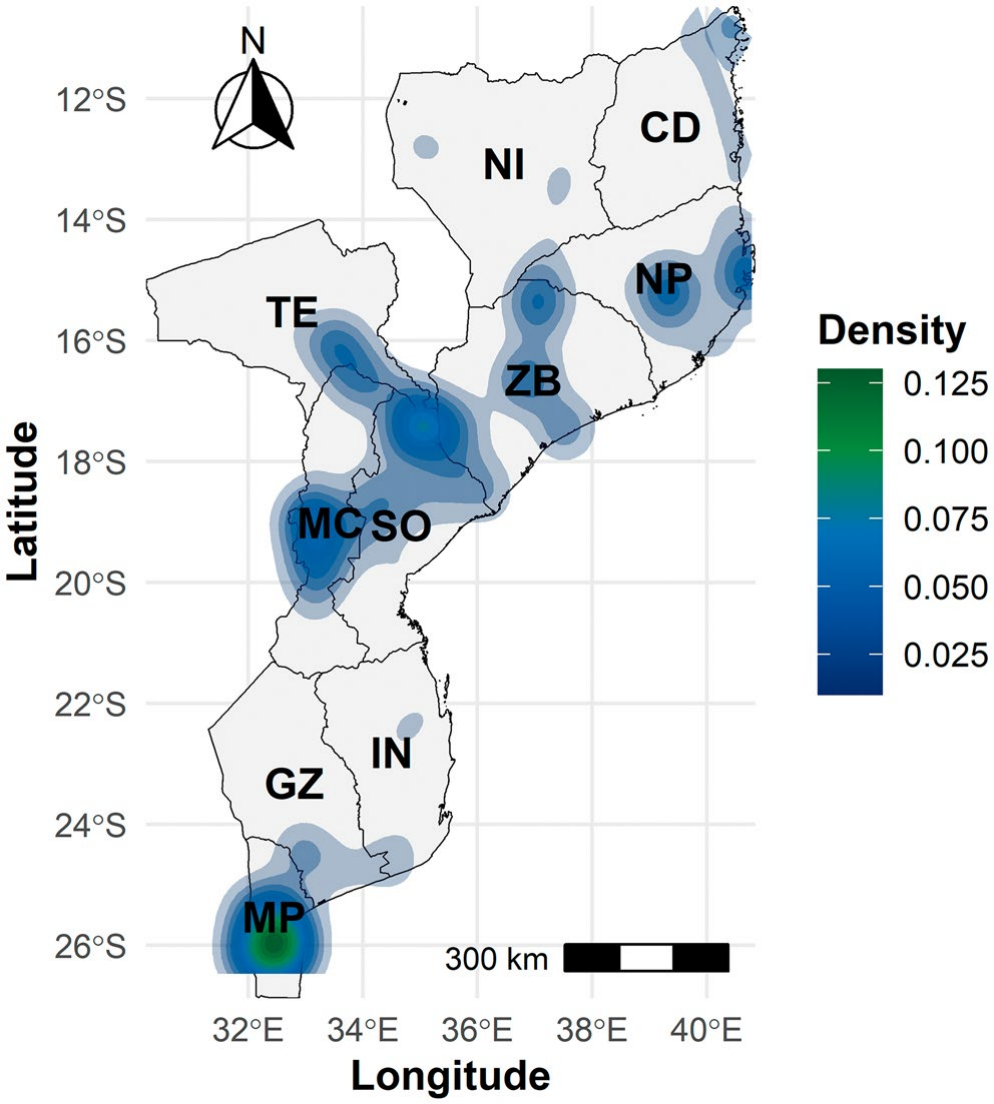
Kernel Density Estimation (KDE) of collection effort, highlighting areas with higher sampling intensity. The colour scale denotes density levels, where darker colours correspond to greater specimen concentration. Mozambique provinces: CD, Cabo Delgado; GZ, Gaza; IN, Inhambane; MC, Manica; MP, Maputo; NI, Niassa; NP, Nampula; SO, Sofala; TE, Tete; ZB, Zambezia.

The spatial distribution patterns of type specimens were further assessed using Kernel Density Estimation (KDE), as presented in Figure 4. This analysis reveals collection density hotspots predominantly in the southern and central regions of Mozambique. Maputo stands out as the most prominent hotspot, followed by Manica, Sofala, Zambezia, Tete and Nampula, all showing moderate collection density.

**FIGURE 5:**
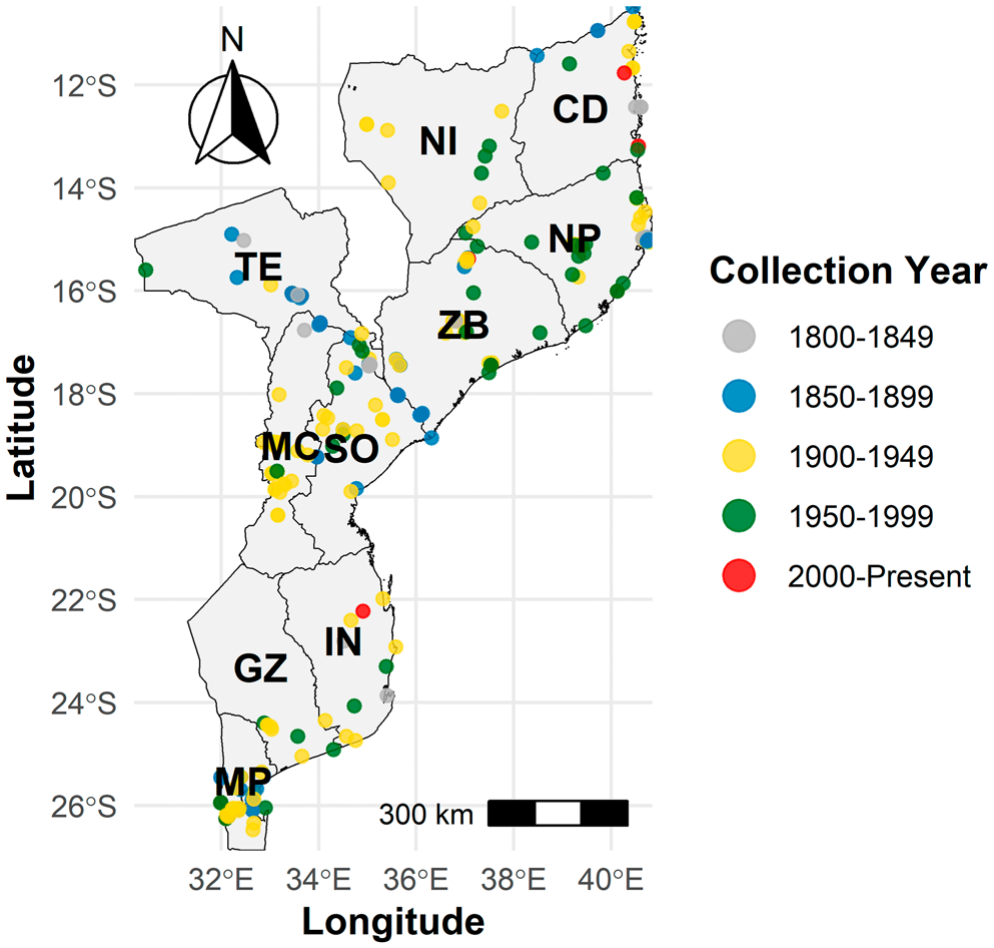
Spatial distribution of Fabaceae type specimens collected in Mozambique. Coloured points represent specimen collection sites, grouped by 50‐year periods and distinguished by colours. Mozambique provinces: CD, Cabo Delgado; GZ, Gaza; IN, Inhambane; MC, Manica; MP, Maputo; NI, Niassa; NP, Nampula; SO, Sofala; TE, Tete; ZB, Zambezia.

Figure 5 illustrates the temporal trends in type specimen collection across 50‐year intervals, highlighting a concentration of collection activity during the 1900s. Early collections (1800s and 1850s) primarily took place along the coastal regions and across the Zambezi River, particularly in Sofala, Manica and Tete. Throughout the 1900s, the collections were unevenly distributed across the country, with the highest concentration in Manica, Maputo and Sofala. Similar patterns emerged in the 1950s, but with increased prominence in the northeastern provinces of Niassa, Nampula and Zambezia. In contrast, during the 2000s, collection activity declined significantly, with only sparse records from Cabo Delgado, Inhambane and Zambezia, despite a general resurgence in overall botanical collecting in Mozambique through various projects.

**FIGURE 6:**
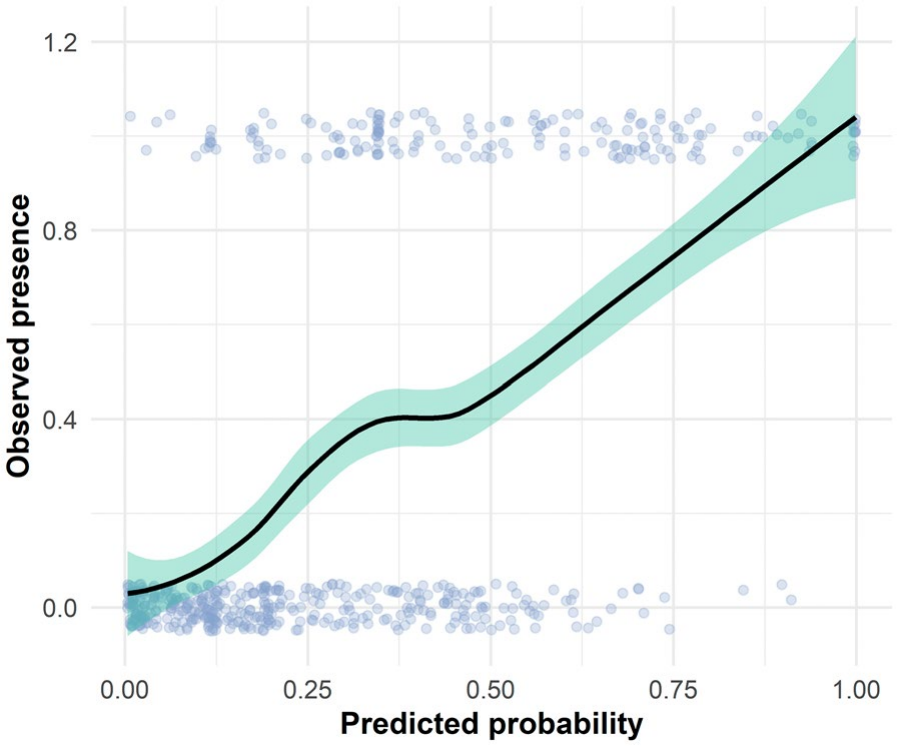
Predicted probability of presence vs. observed occurrences. Predicted probabilities of Fabaceae type specimen presence (from the best‐fit binomial GLM) are plotted on the x‐axis, while observed presence (1) or absence (0) is shown on the y‐axis. Points were jittered vertically to avoid overplotting. The LOESS‐smoothed curve illustrates the logistic pattern captured by the model, confirming an increasing likelihood of presence with higher predicted values. This supports the adequacy of the model in describing the spatial pattern of specimen collection.

The GLM that incorporated elevation, slope, land cover and distances to the nearest roads and harbours provided the best fit for our data (Table 3), significantly outperforming alternative models that considered subsets of predictor variables (Table S3). Among the predictors, elevation, slope and land cover had significant positive effects, indicating a higher probability of specimen collection in higher‐altitude, steeper terrains within specific land cover types. Despite this, most specimens were collected at low altitudes (< 150 m) and on soft slopes (< 2°) (Figure S2). This reflects that a smaller subset of records from montane areas, such as the Chimanimani Mountains, disproportionately influenced the model estimates for these predictors. Conversely, distances to the nearest roads and harbours showed strong negative effects, reflecting collection biases towards more accessible locations (Table 3). The full model displayed the lowest Akaike Information Criterion (AICc = 586.92) and the highest Pseudo *R* ^2^ (0.55), confirming its superior explanatory power compared to reduced models using only biogeographical or accessibility variables (Table S3). Furthermore, the model demonstrated good predictive performance, with an AUC of 0.778, indicating a reliable ability to distinguish between the presence and pseudo‐absence of type specimens (Figure S3). Figure 6 presents the GLM‐fitted response curve, demonstrating how the probability of type specimen collection varies across combinations of predictor variables.

**TABLE 3:** Generalised Linear Model (GLM) results for predictors of Fabaceae type specimens' presence in Mozambique.

Predictor variable	Coefficient estimate	Standard error	*z* value	*p*
Intercept	0.4060	0.3587	1.132	0.258
Elevation (m)	0.0013	0.00054	2.387	0.017*
Slope (°)	0.8657	0.2949	2.949	0.003**
LULC (categorical)	0.2195	0.1066	2.059	0.039*
Distance to the nearest road (m)	−0.000044	0.000011	−3.959	< 0.001***
Distance to the nearest harbour (m)	−0.000007	0.000001	−7.616	< 0.001***

*Note:* Significance codes: ***p < 0.001, **p < 0.01, *p < 0.05.

## 4 Discussion

This study presents the first systematic analysis focused on the discovery and description of the Fabaceae family in Mozambique, offering insights into the taxonomic, temporal and spatial patterns that influence botanical exploration. Our results indicate clear biases in specimen collection, driven by factors such as accessibility and historical floristic initiatives, which have direct implications for understanding plant biodiversity and conservation planning. These findings also highlight significant gaps in the botanical documentation of the country's flora and underscore the development of guidelines for future inventory, taxonomy and conservation efforts. In fact, Stropp et al. (2016), using data from 300 years of angiosperm collections across Africa, identified regions of Central Africa, South Africa and Mozambique as ‘biodiversity blindspots’, where the scarcity of specimen collections hinders the understanding of plant diversity. For instance, a recent study of historical collections from Mozambique revealed that among more tham 300 specimens collected by Américo Pires de Lima between 1916 and 1917 and housed in the Herbarium of the University of Porto (PO), around 40% remain unidentified due to the lack of specialist revision; of the 17 species he described as new to science, only two are currently accepted (Faria et al. 2024). Such historical gaps highlight the long‐standing nature of under‐collection in certain tropical regions and reinforce its impact on taxonomic knowledge and conservation planning.

A detailed analysis of the Fabaceae type specimens collected in Mozambique revealed significant taxonomic diversity, with 126 currently accepted taxa, representing 65% of the 273 identified specimens; the remaining 35% are currently considered taxonomic synonyms. Although excluded from the diversity analysis, these synonyms retain considerable scientific and historical value (Funk et al. 2005; Turland et al. 2018). Among the accepted taxa, the genera *Indigofera*, *Crotalaria* and *Tephrosia* predominated. These genera are among the most species‐rich within Fabaceae in Mozambique (Mackinder et al. 2001; Pope et al. 2003; Schrire 2012; Odorico et al. 2022; Hyde et al. 2025) and across tropical Africa (Schrire 2012; Catarino et al. 2022), possibly reflecting both natural diversity patterns and collection‐related biases. The high proportion of endemic (35%) and near‐endemic (14%) taxa—expected, as type specimens originate from Mozambique—highlights Mozambique's importance as a plant endemism hotspot (Darbyshire et al. 2019). The predominance of woody species (55%), particularly shrubs and trees, is consistent with the vegetation structure of savannas, open forests and dry woodlands typical of Mozambique (White 1983; Burrows et al. 2018). This pattern likely reflects a historical and ongoing bias towards woody flora, stemming from colonial‐era forestry priorities and reinforced by national forest surveys and focused botanical exploration (Burrows et al. 2018). Although smaller species are generally more accessible and often more frequently collected, trees and lianas tend to be underrepresented due to the practical challenges involved in sampling and specimen preparation (Daru et al. 2018). This apparent contradiction may therefore result from targeted taxonomic interest in woody taxa combined with uneven sampling across habitats.

Our data showed that only 56% of the accepted taxa have been assessed on the IUCN Red List of Threatened (IUCN 2025), with 19% classified as threatened (i.e., VU, EN, CR), reinforcing the urgency of focused conservation measures. While collector bias may lead to an overrepresentation of certain rare species, as some collectors aim to maximise species diversity (Steege et al. 2011), many threatened species remain underrepresented in herbarium collections due to their rarity and legal or ethical restrictions on collection. These limitations, well documented in recent studies (Verspagen and Erkens 2023), constrain our understanding of species distributions and conservation needs, potentially leading to biased extinction risk assessments (Nic Lughadha et al. 2020). Moreover, our data show that seven endemic Fabaceae taxa are known only from their type specimen, exhibiting a pattern also described by Darbyshire et al. (2019), who reported that almost a quarter of Mozambique's strict endemics are similarly restricted in documentation. It is important to interpret these findings with caution, as they reflect only a subset of Fabaceae diversity in Mozambique, namely, the type specimens, whose primary role is nomenclatural (Turland et al. 2018). Although these specimens provide essential taxonomic references, they represent only a narrow fraction of collections and do not capture the full ecological spectrum or intraspecific variation, limiting their direct use in conservation planning (Meyer et al. 2015). Moreover, since herbarium specimens are key to documenting Fabaceae diversity, the use of expert‐curated checklists enhances the accuracy of occurrence records and taxonomic identifications, supporting robust biodiversity analyses (le Roux et al. 2022). Therefore, integrating type specimens data with field‐based inventories and further taxonomic and ecological studies is essential to support more effective conservation decisions.

At a temporal level, our data indicate four main historical peaks of intensified collecting efforts, which occurred in the 1860s, 1890s, 1940s and 1960s. The number of taxa published over time follows the trend of specimen collection, with the highest peaks of taxonomic output observed in the 1950s and 1960s. These patterns coincide with the periods when major regional floristic programmes were active: the Flora of Tropical Africa (1868–1937), the Flora of Tropical East Africa (1952–2012) and the Flora Zambesiaca (1960‐present), which not only structured regional botanical exploration but also proved to be fundamental in the discovery and documentation of Mozambique's plant diversity (Beentje 2016; Darbyshire et al. 2019). In this sense, the temporal trajectory of species publication can be viewed in three main phases: treatments in the Flora of Tropical Africa mainly between 1871 and 1930; in the Flora of Tropical East Africa, Mimosoideae and Caesalpinoideae in 1967 and Papilionoideae in 1971 and in the Flora Zambesiaca, Mimosoideae in 1970, Caesalpinoideae in 2007 and Papilionoideae across multiple parts between 1998 and 2012. This alignment between collection intensity and publication highlights how coordinated flora projects and targeted fieldwork shaped our understanding of Mozambique's Fabaceae diversity.

Several important botanical expeditions took place in the country and contributed to the aforementioned floristic programmes. For example, David Livingstone's Zambezi Expedition, between 1858 and 1864, represented one of the first milestones in the discovery of the Fabaceae family in Mozambique, during which John Kirk collected around 1400 herbarium specimens (Liberato 1994; Goyder 2022). From the late 19th century to the mid‐20th century, the Portuguese colonial context, driven by European scientific institutions, played a leading role in expanding botanical knowledge of the Mozambican flora (Figueiredo and Smith 2010). Within this context, the ‘Botanic Mission to Mozambique’ stands out, a systematic campaign promoted by the ‘Junta das Missões Geográficas e de Investigações Coloniais’, created in 1936 by the Portuguese government to integrate scientific knowledge into colonial policy and to draw up the Phytogeographic Map of Mozambique for the Atlas of the Colonial Empire (Castelo 2012). Conducted over three expeditions between 1942 and 1948, the mission gathered approximately 7600 herbarium specimens and contributed to the description of several new species (Conde et al. 2014). The 1960s and 1970s marked another intense period of specimen collection, coinciding with the first fascicles of Flora Zambesiaca, which was later interrupted by independence and civil wars (Martins and Duarte 2010). Darbyshire et al. (2019) similarly reported a surge in the description of Mozambique's endemics after 1950, with the mean year of first publication being 1950 (or 1967 for strict endemics), demonstrating how coordinated flora projects and associated fieldwork unlocked major taxonomic advances. After a prolonged period of instability, botanical exploration resumed from the 2000s onwards (Cheek et al. 2018), leading to significant progress such as the publication of the most recent checklist of Mozambique flora (Odorico et al. 2022) and the identification of a national network of Important Plant Areas (Darbyshire et al. 2023). Nonetheless, major knowledge gaps persist: nearly one quarter of Mozambique's strict endemics are still known only from their type specimens or type locality (Darbyshire et al. 2019), a trend also observed in Fabaceae, where approximately 15% of strict endemics are represented solely by type collections.

A close alignment between the number of newly described taxa and collection intensity suggests that taxonomic progress is heavily dependent on concentrated fieldwork efforts, as expected. However, an average time lag of 18 years between specimen collection and formal description was observed, revealing that several new species remained undescribed for decades. This pattern aligns with global analyses by Bebber et al. (2010), who demonstrated that a significant proportion of newly described plant taxa are based on herbarium specimens that remained unrecognised for decades before formal description. Such delays illustrate the Linnaean shortfall (Hortal et al. 2015), emphasising that many vascular plants remain scientifically undescribed even after being collected and stored in herbaria (Joppa et al. 2011). A remarkable example is *Icuria dunensis* Wieringa, a large, habitat‐forming tree strictly endemic to the coastal dry forests of northern Mozambique, which was only described as a new genus in 1999 despite being represented by earlier, misidentified collections (Wieringa 1999). This case highlights the extent to which parts of the Mozambican flora have been overlooked until recently. Similarly, recent expeditions to Cabo Delgado have led to the publication of several new species from the coastal dry forests and woodlands of the Rovuma Centre of Endemism, demonstrating ongoing progress in addressing these knowledge gaps (Darbyshire et al. 2020). This result also underscores the crucial, yet often neglected, role of herbaria in biodiversity discovery. The gap between specimen availability and taxonomic capacity has critical implications for both conservation and scientific understanding, as it delays the recognition of potentially rare or endangered species and hinders timely policy responses (Carine et al. 2018).

Our results indicate that a small group of collectors made disproportionate contributions to the knowledge of the Fabaceae family in Mozambique from the mid‐19th century to the end of the 20th century. Only ten collectors were responsible for over 70% of the Fabaceae type specimens' collections. This pattern is recognised globally, as described by Bebber et al. (2012), who reported that more than half of all type specimens were collected by < 2% of collectors. Furthermore, Romeiras et al. (2020) found that only 19% of collectors were responsible for collecting more than 60% of the type specimens of endemic species in the Cabo Verde archipelago. It should be noted that collectors do not gather type specimens intentionally. All plant material is collected as ordinary specimens, and only later, when a taxon is formally described, may one of these be designated as holotype, isotype or other type in accordance with the International Code of Nomenclature (Turland et al. 2018). Consequently, the attribution of type material to particular collectors is retrospective, reflecting taxonomic decisions made years or even decades after the original collection. Acknowledging this distinction is essential for interpreting patterns in collector contribution and the temporal lag between specimen collection and publication.

Furthermore, the number of type specimens attributed to a collector is strongly influenced by the total volume of material they have gathered. This means that collectors perceived as important often owe this status to prolific collecting activity rather than a targeted search for new taxa. Many type specimens were described long after collection by different taxonomists, so the association between collectors and newly described species is largely a consequence of subsequent systematic work rather than intentional discovery. This distinction is crucial for interpreting collector significance, and future comparisons between total collecting effort and the number of new taxa described from those specimens would provide further insights.

Among the most prolific collectors—for example, António Rocha da Torre, John Kirk, Wilhelm Peters and Luis Grandvaux Barbosa—specimens were collected in multiple provinces and over several decades, significantly contributing to both taxonomic and geographical coverage. This strategy aligns with common practices aimed at increasing taxonomic representation by sampling across diverse locations (Schmidt et al. 2025). Furthermore, we found a significant correlation between the duration of a collector's activity and the number of type specimens collected, suggesting that sustained individual efforts have a direct impact on new taxonomic discoveries. This tendency is supported by Bebber et al. (2012), who demonstrated that productivity in collecting new types increases with the time spent in the field, although the relationship is nonlinear.

The spatial distribution of the Fabaceae type specimens exhibited distinct collection patterns, with higher concentrations in the central and southern provinces (i.e., Maputo, Sofala, Zambezia, Manica and Nampula), while northern and inland regions, such as Niassa, Cabo Delgado and Tete, remain sampled. Collections were heavily clustered near roads, harbours, rivers and coastal zones, with Kernel density estimation confirming hotspots around urban centres, major transport routes and the coastline. This trend echoes global observations that herbarium records are denser in areas with better logistical support and proximity to research institutions (Daru and Rodriguez 2023). While this pattern largely reflects human accessibility bias, the prominence of coastal areas also indicates a positive bias, as Mozambique coastal vegetation is exceptionally rich in endemism and has therefore been a specific target for biodiversity surveys, contrasting with much of the interior, which is relatively depauperate in endemism (Darbyshire et al. 2019). As noted by Romeiras et al. (2014), collections have often favoured accessible and well‐known areas; remote areas in Angola, for instance, were largely overlooked, and a similar pattern likely applies to isolated provinces such as Gaza and Niassa. Such a spatial bias illustrates the Wallacean shortfall, which refers to gaps in knowledge about species distributions due to uneven sampling (Hortal et al. 2015; Ondo et al. 2024). These disparities may help explain the underrepresentation of type specimens in certain Mozambican regions.

Beyond the influence of accessibility and topography, the spatial distribution of Fabaceae type specimens reveals strong associations with biodiversity‐relevant areas of ecological and conservation significance. About 13% of collections were recorded within Important Plant Areas (IPAs), despite these areas covering just 3% of the national territory (Darbyshire et al. 2023), highlighting their important conservation value. Notably, around 60% of type specimens originate from recognised centres of endemism, with the Rovuma centre alone accounting for 19%. This centre is particularly distinguished by its high number of strict endemics, reflecting the pronounced species turnover among the fragmented dry coastal forest patches characteristic of the region (Timberlake et al. 2011), where many species are restricted to only a few or even single forest blocks. Furthermore, about 70% of type specimens were collected within coastal ecoregions, specifically Dry miombo woodlands, Maputaland coastal forest and woodlands and Southern Swahili coastal forest and woodlands. These patterns confirm that type specimen concentration is driven not only by accessibility but also by the rich endemic and ecological diversity of Mozambique's coastal zones. This bias highlights the need for targeted conservation measures in coastal and endemic‐rich regions, while simultaneously addressing substantial sampling gaps in inland and less accessible areas.

Our GLM confirmed that proximity to nearest roads and harbours shows significant negative associations, indicating that the probability of specimen collection decreases as distance from these features increases. This emphasises a clear bias in collections towards more accessible areas. Conversely, environmental factors such as altitude and slope showed positive effects, which may seem contradictory given that most specimens were collected at low altitudes (< 150 m) and on soft slopes (< 2°). This apparent contraction likely arises from focused collecting in montane areas characterised by high plant diversity and endemism, such as Chimanimani Mountains and Mount Namuli, which elevate the probability of sampling in steeper and higher‐altitude regions, despite most collections occurring at lower elevation and flatter areas. For instance, Catarino et al. (2022) reported higher endemic Fabaceae collection at greater altitudes in Angola's Serra de Chela. In Mozambique, our mapping confirmed several new Fabaceae species collected in the Chimanimani Mountains, a region previously recognised for high plant diversity and endemism (Timberlake et al. 2016; Wursten et al. 2017). Conversely, the southern lowlands and certain interior regions show sparse records, which could reflect either reduced sampling effort or indeed lower plant diversity, a distinction that is challenging to disentangle without species richness modelling. For example, provinces such as Gaza and Inhambane are among those with the lowest numbers of recognised plant species in Mozambique (Odorico et al. 2022).

A key limitation of this study is the temporal mismatch between historical specimen records and contemporary LULC and accessibility data, as landscapes and infrastructures have changed over time. Although contemporary spatial data present limitations, incorporating habitat and site descriptions from herbarium specimen labels, when available, provides valuable context to interpret collection patterns more accurately. This challenge is common in ecological studies relying on herbarium data when historical spatial datasets are lacking (Brilhante et al. 2025). Such limitations should be considered when interpreting results and planning future research.

To address the taxonomic and spatial shortfalls described by our approach, it is essential to adopt integrated strategies that combine targeted botanical surveys in under‐sampled regions, especially northern and southern inland provinces, with investment in research infrastructures, such as the expansion of national herbaria, taxonomic training programmes and international partnerships with botanical institutions. Strengthening the role of local collectors is also important for building long‐term taxonomic capacity. Continued efforts in digitising, curating and georeferencing collections will further improve data accessibility and support spatial analyses critical for conservation (Eckert et al. 2024). Such actions are essential to mitigating both Linnaean and Wallacean shortfalls (Hortal et al. 2015), which still limit our understanding of the scientific identity and geographical distribution of species.

In summary, the analysis of Fabaceae type specimen records in Mozambique reflects a complex interplay of historical, environmental and sociopolitical factors. Colonial expeditions, wars and post‐independence instability have all left visible imprints on the patterns and rate of plant collection and discovery in the country. Moreover, accessibility has also played a preponderant role in collection efforts through time. Recognising and understanding these biases is crucial to inform future initiatives aimed at expanding and refining the documentation and preservation of the national flora. Such efforts will contribute both to the preservation of Mozambique's biodiversity and its broader scientific and cultural valorisation.

## Author Contributions

**Miguel Brilhante:** conceptualization (equal), investigation (equal), methodology (equal), writing – original draft (equal), writing – review and editing (equal). **Iain Darbyshire:** investigation (equal), writing – review and editing (equal). **Maria Cristina Duarte:** investigation (equal), writing – review and editing (equal). **Margarida Moldão:** supervision (equal), writing – review and editing (equal). **Salomão Bandeira:** investigation (equal), supervision (equal), writing – review and editing (equal). **Maria M. Romeiras:** conceptualization (equal), investigation (equal), supervision (equal), writing – review and editing (equal).

## Funding

This work was supported by Fundação para a Ciência e a Tecnologia (FCT), UI/BD/151188/2021 to Miguel Brilhante; UIDB/04129/2025—https://doi.org/10.54499/UID/04129/2025 (Linking Landscape, Environment, Agriculture and Food—LEAF); and UID/00329/2025—https://doi.org/10.54499/UID/00329/2025 (Centre for Ecology, Evolution and Environmental Changes—CE3C).

## Conflicts of Interest

The authors declare no conflicts of interest.

## Supporting information


**Appendix S1:** ece372854‐sup‐0001‐AppendixS1.docx.

## Data Availability

The curated dataset (Table S1), listing Fabaceae type specimens collected in Mozambique and including taxonomic and ecological attributes, has been deposited in Zenodo and is publicly available at https://doi.org/10.5281/zenodo.17209081. All data supporting the findings of this study are available within the paper and its Supporting Information.
