# Supplementary material for: Mapping Biodiversity Through Time and Space: Patterns and Drivers of Fabaceae Collection in Mozambique
Source: Ecol Evol. 2026 Feb 17;16(2):e72854. doi: 10.1002/ece3.72854 (PMC12912884; doi:10.1002/ece3.72854)
Supplement: Supplementary file 1 — Appendix S1: ece372854‐sup‐0001‐AppendixS1.docx. [file ECE3-16-e72854-s001.docx]

Title: **Mapping Biodiversity Through Time and Space – Patterns and Drivers of Fabaceae Collection in Mozambique**

Miguel Brilhante^1^, Iain Darbyshire^2^, Maria Cristina Duarte^3^, Margarida Moldão^1,4^, Salomão Bandeira^5^, Maria M. Romeiras^1,3,4*^

^1^Linking Landscape, Environment, Agriculture and Food (LEAF), Instituto Superior de Agronomia (ISA), Universidade de Lisboa, Tapada da Ajuda, 1340-017 Lisboa, Portugal

^2^Royal Botanic Gardens, Kew, Richmond, TW9 3AE, United Kingdom

^3^Centre for Ecology, Evolution and Environmental Changes (CE3C) and Change–Global Change and Sustainability Institute, Faculdade de Ciências, Universidade de Lisboa, 1749-016 Lisboa, Portugal

^4^Associate Laboratory TERRA, Instituto Superior de Agronomia (ISA), Universidade de Lisboa, Tapada da Ajuda, 1340-017 Lisboa, Portugal

^5^Department of Biological Sciences, Eduardo Mondlane University, PO Box 257, Maputo 1100, Mozambique

*Corresponding author: Maria Manuel Romeiras, Linking Landscape, Environment, Agriculture and Food (LEAF), Associate Laboratory TERRA, Instituto Superior de Agronomia (ISA), Universidade de Lisboa, Tapada da Ajuda, 1340-017 Lisboa, Portugal. E-mail: [mmromeiras@isa.ulisboa.pt](mailto:mmromeiras@isa.ulisboa.pt)

Running title: **Patterns of Fabaceae Collection in Mozambique**

**Table S1.** List of the Fabaceae type specimens collected in Mozambique, including details on the native status, growth form, IUCN conservation status, native distribution, type collector name, type collection location, geographical coordinates of the collection location, year of collection and host herbarium, and protologue.

| **Scientific Name^1^ [=accepted name]** | **Native Status^2^** | **Growth-form** | **IUCN ^3^** | **Native Distribution** | **Protologue** | **Type Collector** | **Type Location** | **Latitude** | **Longitude** | **Type Collection Year** | **Only known from type** | **Type and Host Herbarium** |
| --- | --- | --- | --- | --- | --- | --- | --- | --- | --- | --- | --- | --- |
| *Abrus gracilis* Pires de Lima [=*Abrus melanospermus* Hassk. subsp. *tenuiflorus* (Spruce ex Benth.) D.K.Harder] |  |  |  |  | Brotéria, Sér. Bot. 19: 127 (1921) | Pires de Lima 257 | Cabo Delgado, surroundings of Palma | -10.7758 | 40.4673 | 1917 |  | Holotype (PO) |
| *Abrus tunguensis* Pires de Lima [=*Abrus precatorius* L. subsp. *africanus* Verdc.] |  |  |  |  | Brotéria, Sér. Bot. 19: 127 (1921) | Pires de Lima 94, 134 | Cabo Delgado, surroundings of Palma | -10.7758 | 40.4673 | 1917 |  | Holotype (PO) |
| ***Acacia adenocalyx* Brenan & Exell [=*Senegalia adenocalyx* (Brenan & Exell) Kyal. & Boatwr.]** | Native | Shrub or subshrub | LC | S. Kenya to N. & Central Mozambique | Bol. Soc. Brot., sér. 2, 31: 115 (1957) | Andrada 1097 | Sofala, Cheringoma, between Inhaminga and Inhamitanga | -18.4996 | 35.3014 | 1948 |  | Paratype (LISC) |
|  |  |  |  |  | Bol. Soc. Brot., sér. 2, 31: 115 (1957) | Barbosa & Correia 2417 | Nampula, Nacala, surroundings of Fernão Veloso | -14.4642 | 40.6899 | 1948 |  | Paratype (LISC) |
|  |  |  |  |  | Bol. Soc. Brot., sér. 2, 31: 115 (1957) | Torre 1431 | Nampula, Memba, Nacala road | -14.2036 | 40.5235 | 1937 |  | Paratype (LISC) |
| ***Acacia borleae* Burtt Davy [=*Vachellia borleae* (Burtt Davy) Kyal. & Boatwr.]** | Native | Shrub or subshrub | LC | SE. Zimbabwe, S. Mozambique to S. Africa | Bull. Misc. Inform. Kew 1922: 325 (1922) | Borle 271 | Maputo | -25.4486 | 32.3984 | 1920 |  | Holotype (PRE) |
| *Acacia caffra* var. *rupestris* Sim [=*Senegalia afra* (Thunb.) P.J.H.Hurter & Mabb.] |  |  |  |  | Forest Fl. Port. E. Afr.: 56 (1909) | Sim 6235 | Maputo, Marracuene and Maputo | -25.8694 | 32.6611 | 1908 |  | Not traced |
| *Acacia delagoensis* Harms [=*Senegalia welwitschii* (Oliv.) Kyal. & Boatwr. subsp. *delagoensis* (Harms) Kyal. & Boatwr.] |  |  |  |  | Bot. Jahrb. Syst. 51: 367 (1914) | Schlechter 11718 | Maputo, Umbuluzi | -26.0774 | 32.3562 | 1897 |  | Isotype (K) |
| ***Acacia latispina* J.E.Burrows & S.M.Burrows [=*Vachellia latispina* (J.E.Burrows & S.M.Burrows) Kyal. & Boatwr.]** | Strict-Endemic | Tree | VU | Mozambique | Bothalia 39(2): 222 (-224; figs. 3-4, map) (2009) | Burrows & Burrows 9764 | Cabo Delgado Province, 14.8 km from the main Pemba-Metoro road, on road to Mecufi | -13.1869 | 40.5528 | 2006 |  | Holotype (PRE) |
| *Acacia mossambicensis* Bolle [=*Faidherbia albida* (Delile) A.Chev.] |  |  |  |  | W.C.H.Peters, Naturw. Reise Mossambique 6(Bot., 1): 15 (1861) | Peters s.n. | Tete, Rios de Sena and R. Chimzao, W. of Tete | -15.0215 | 32.4556 | Unknown |  | Syntype (B)* |
| *Acacia nigrescens* var. *pallens* Benth. [=*Senegalia nigrescens* (Oliv.) P.J.H.Hurter] |  |  |  |  | Trans. Linn. Soc. London 30: 517 (1875) | Kirk 201 | Tete, near Sena | -17.4317 | 35.0260 | 1860 |  | Holotype (K) |
| ***Acacia quiterajoensis* Timberlake & Lötter** | Strict-Endemic | Shrub or subshrub | LC | Mozambique | Trees Shrubs Mozambique: 1041 (2018) | Timberlake, Luke, Crawford & Goyder 5795 | Cabo Delgado, Macomia, Quiterajo area, Goba village | -11.7639 | 40.2694 | 2009 |  | Holotype (K) |
| ***Acacia retinens* Sim [=*Vachellia luederitzii* (Engl.) Kyal. & Boatwr. var. *retinens* (Sim) Kyal. & Boatwr.]** | Native | Shrub or subshrub | NE^4^ | Mozambique to S. Africa | Forest Fl. Port. E. Afr.: 57 (1909) | Sim 6391 | Maputo, Umbuluzi and Lebombo | -26.0515 | 32.2440 | 1908 |  | Not traced |
| *Acacia rostrata* Sim [=*Senegalia senegal* (L.) Britton] |  |  |  |  | Forest Fl. Port. E. Afr.: 55 (1909), nom. illeg. | Sim 6263 | Maputo | -25.4486 | 32.3984 | 1908 |  | Not traced |
| ***Acacia rovumae* Oliv. [=*Senegalia rovumae* (Oliv.) Kyal. & Boatwr.]** | Native | Tree | LC | S. Somalia to N. Mozambique, Madagascar | Fl. Trop. Afr. 2: 353 (1871) | Kirk s.n. | Cabo Delgado, Rovuma Bay | -10.4791 | 40.4457 | 1861 |  | Holotype (K) |
| *Acacia sambesiaca* Schinz [=*Vachellia robusta* (Burch.) Kyal. & Boatwr. subsp. *clavigera* (E.Mey.) Kyal. & Boatwr.] |  |  |  |  | Denkschr. Kaiserl. Akad. Wiss., Wien. Math.-Naturwiss. Kl. 78: 416 (1908) | Menyhart 1003 | Zambézia, Boroma | -17.4451 | 35.6600 | 1891 |  | Holotype (WU) |
| ***Acacia schlechteri* Harms [=*Senegalia schlechteri* (Harms) Kyal. & Boatwr.]** | Strict-Endemic | Tree | DD | Mozambique (Ressano Garcia) | Bot. Jahrb. Syst. 51: 367 (1914) | Schlechter 11901 | Maputo, Ressano Garcia | -25.4431 | 31.9924 | 1897 |  | Isotype (K) |
| ***Acacia schweinfurthii* Brenan & Exell [=*Senegalia schweinfurthii* (Brenan & Exell) Seigler & Ebinger]** | Native | Woody climber | LC | South Sudan to S. Africa | Bol. Soc. Brot. sér. 2, 31: 128 (1957) | Andrada 1055 | Sofala, Cheringoma, between Inhaminga and Inhamitanga | -18.4996 | 35.3014 | 1948 |  | Paratype (LISC) |
|  |  |  |  |  | Bol. Soc. Brot. sér. 2, 31: 128 (1957) | Garcia 934 | Manica, On the banks of the river Muda. Lamego region. Between Vila Peri and Macequece | -19.0120 | 33.0968 | 1948 |  | Paratype (LISC) |
|  |  |  |  |  | Bol. Soc. Brot. sér. 2, 31: 128 (1957) | Torre 3898 | Gaza, in the dense bush of the marginal plains of the Limpopo River. Near Vila Joao (Xai-Xai) | -25.0383 | 33.6515 | 1942 |  | Paratype (LISC) |
|  |  |  |  |  | Bol. Soc. Brot. sér. 2, 31: 128 (1957) | Torre 2136 | Maputo, Salamanga, on the banks of Maputo river | -26.4714 | 32.6497 | 1940 |  | Paratype (LISC) |
| ***Acacia torrei* Brenan [=*Vachellia torrei* (Brenan) Kyal. & Boatwr.]** | Strict-Endemic | Shrub or subshrub | LC | Mozambique | Kew Bull. 21: 480 (1968) | Torre 4068 | Sofala, between Inhaminga and Rio Urema | -18.7147 | 34.7807 | 1942 |  | Holotype (LISC) |
| ***Acacia xanthophloea* Benth. [=*Vachellia xanthophloea* (Benth.) Banfi & Galasso]** | Native | Tree | LC | N. Somalia to S. Africa | Trans. Linn. Soc. London 30: 511 (1875) | Kirk s.n. | Sofala, Vila de Sena | -17.4525 | 35.0325 | 1860 |  | Syntype (K) |
| ***Aeschynomene chimanimaniensis* Verdc.** | Near-Endemic | Shrub or subshrub | LC | Zimbabwe to Mozambique | Kew Bull. 27: 435 (1972) | Wild 2888 | Manica, Chimanimani Mts. | -19.8667 | 33.1000 | 1949 |  | Holotype (K) |
| ***Aeschynomene grandistipulata* Harms** | Near-Endemic | Shrub or subshrub | LC | S. Tropical Africa | Repert. Spec. Nov. Regni Veg. 8: 355 (1910) | Johnson 232 | Manica, Chimanimani Mts., Moribane | -19.9167 | 33.2000 | 1907 |  | Holotype (K) |
| ***Aeschynomene inyangensis* Wild** | Near-Endemic | Shrub or subshrub | LC | S. Tropical Africa | Kew Bull. 8: 93 (1953) | Torre 5849 | Manica, Bárui, Serra de Choa, Colinas da serra. | -18.0170 | 33.1863 | 1943 |  | Paratype (LISC) |
| ***Aeschynomene minutiflora* Taub. subsp. *grandiflora* Verdc.** | Strict-Endemic | Annual or biennial herb | NE | Mozambique | Kew Bull. 27: 437 (1972) | Balsinhas & Marrime 325 | Nampula, Eráti, Namapa, near the Experimental Station CICA, on path to River Lúrio | -13.7122 | 39.8321 | 1961 |  | Holotype (K) |
|  |  |  |  |  | Kew Bull. 27: 437 (1972) | Torre 815 | Nampula, surroundings of Nampula | -15.1091 | 39.2698 | 1936 |  | Paratype (LISC) |
|  |  |  |  |  | Kew Bull. 27: 437 (1972) | Torre 1216 | Nampula, surroundings of Nampula | -15.1091 | 39.2698 | 1937 |  | Paratype (LISC) |
|  |  |  |  |  | Kew Bull. 27: 437 (1972) | Torre & Correia 15004 | Zambezia, Ile, Errego, ca. 3 km, Mount Ile | -16.0348 | 37.1705 | 1966 |  | Paratype (LISC) |
|  |  |  |  |  | Kew Bull. 27: 437 (1972) | Torre & Paiva 11648 | Cabo Delgado, Mecúfi, 15 km from Namapa to Porto Amélia | -13.2558 | 40.5351 | 1964 |  | Paratype (LISC) |
|  |  |  |  |  | Kew Bull. 27: 437 (1972) | Torre & Paiva 11553 | Nampula, 21 km from Nampula to Nametil | -15.3290 | 39.3326 | 1964 |  | Paratype (LISC) |
| ***Aeschynomene mossambicensis* Verdc. subsp. *mossambicensis*** | Strict-Endemic | Annual or biennial herb | NE | Mozambique | Kew Bull. 27: 437 (1972) | Faulkner 206 | Zambezia, Quelimane, Lugela-Mocuba, Namagoa Estate | -16.5909 | 36.8857 | 1946 |  | Holotype (K) |
|  |  |  |  |  | Kew Bull. 27: 437 (1972) | Torre 5208 | Zambézia, surroundings of Maganja da Costa | -17.3931 | 37.5584 | 1943 |  | Paratype (LISC) |
|  |  |  |  |  | Kew Bull. 27: 437 (1972) | Torre 1362 | Nampula, Mogovolas, Nametil | -15.7298 | 39.3201 | 1937 |  | Paratype (LISC) |
|  |  |  |  |  | Kew Bull. 27: 437 (1972) | Torre & Correia 17182 | Zambézia, Pebane, Mualama, at km 27, road to Nabúri | -16.8102 | 38.5329 | 1968 |  | Paratype (LISC) |
|  |  |  |  |  | Kew Bull. 27: 437 (1972) | Torre & Correia 14452 | Zambezia, Mocuba, at kilometre 13 of the Mocuba-Maganja da Costa road junction | -16.7990 | 37.0275 | 1966 |  | Paratype (LISC) |
|  |  |  |  |  | Kew Bull. 27: 437 (1972) | Torre & Paiva 11405 | Nampula, Meconta, 26 km from Corrane to Nampula | -15.2690 | 39.4423 | 1964 |  | Paratype (LISC) |
| ***Aeschynomene schliebenii* Harms** | Native | Shrub or subshrub | NE | Tanzania to S. Tropical Africa | Notizbl. Bot. Gart. Berlin-Dahlem 13: 420 (1936) | Torre 269 | Niassa, Mepoche, Maniamba | -12.7618 | 34.9793 | 1934 |  | Holotype (BM) |
| ***Albizia brevifolia* Schinz** | Native | Shrub or subshrub | LC | S. Tropical & S. Africa | Bull. Herb. Boissier, sér. 2, 2: 945 (1902) | Menyhart 994 | Zambezia, Boroma, on the Nhasinde | -17.4453 | 35.6597 | 1891 |  | Holotype (Z) |
| ***Albizia forbesii* Benth.** | Native | Tree | LC | Tanzania to S. Africa | London J. Bot. 3: 92 (1844) | Forbes s.n. | Maputo, Delagoa Bay | -26.0899 | 32.6398 | 1822 |  | Holotype (K) |
| ***Albizia glabrescens* Oliv. [=*Albizia glaberrima* (Schumach. & Thonn.) Benth. var. *glabrescens* (Oliv.) Brenan]** | Native | Tree | NE^4^ | Kenya to Mozambique, Comoros, Madagascar | Fl. Trop. Afr. 2: 357 (1871) | Kirk s.n. | Sofala, between Tete and the Sea coast | -17.5977 | 34.7392 | 1860 |  | Holotype (K) |
| *Albizia mossambicensis* Sim [=*Albizia versicolor* Welw. ex Oliv.] |  |  |  |  | Forest Fl. Port. E. Afr.: 59 (1909), nom. illeg. | Sim 6392 | Maputo, Umbeluzi, Estação Agronómica | -26.0522 | 32.3616 | 1908 |  | Holotype (NU) |
| *Albizia umbalusiana* Sim [=*Albizia anthelmintica* (A.Rich.) Brongn.] |  |  |  |  | Forest Fl. Port. E. Afr.: 59 (1909) | Sim 6200 | Maputo, up to the Libombo | -26.1894 | 32.1125 | 1908 |  | Not traced |
| *Albizia versicolor* Welw. ex Oliv. var. *mossambicensis* Schinz [=*Albizia versicolor* Welw. ex Oliv.] |  |  |  |  | Bull. Herb. Boissier, sér. 2, 2: 946 (1902) | Menyhart 77b | Zambezia, Boroma | -17.4423 | 35.6535 | 1902 |  | Holotype (Z) |
| *Alysicarpus vaginalis* (L.) A.DC. var. *parvifolius* Verdc. [=*Alysicarpus vaginalis* (L.) A.DC. var. *vaginalis]* |  |  |  |  | Kew Bull. 27: 443 (1972) | Peter s.n. | Nampula, Fortaleza de São Sebastião ("Insel M. des Forts") | -15.0375 | 40.7356 | 1925 |  | Holotype (K) |
| *Anil pentaphylla* var. *trichocarpa* Kuntze [=*Indigofera vohemarensis* Baill.] |  |  |  |  | Revis. Gen. Pl. 3(2): 52 (1898) | Kuntze s.n. | Mozambique |  |  | 1894 |  | Holotype (NY) |
| ***Baphia gomesii* Baker f. [=*Baphia massaiensis* Taub. subsp. *gomesii* (Baker f.) Brummitt]** | Strict-Endemic | Shrub or subshrub | LC | Mozambique | Bol. Soc. Brot., sér. 2, 8: 113 (1933) | Gomes e Sousa 828 | Nampula, Serra de Ribaué | -14.7500 | 37.1667 | 1931 |  | Isotype (LISC) |
| *Baphia mocimboensis* Pires de Lima [=*Baphia macrocalyx* Harms] |  |  |  |  | Brotéria, Sér. Bot. 19: 120 (1921) | Pires de Lima 265 | Cabo Delgado, Mocimboa da Praia, Ponta Vermelha | -11.3478 | 40.3630 | 1917 |  | Holotype (PO) |
| ***Baphia ovata* Sim [=*Baphia kirkii* Baker subsp. *ovata* (Sim) Soladoye]** | Strict-Endemic | Shrub or subshrub | NT | Mozambique | Forest Fl. Port. E. Afr.: 42 (1909) | Sim 5279 | Inhambane, Quisico and occasionally through M'Chopes, usually near water | -24.7315 | 34.7502 | 1908 |  | Holotype (K) |
| ***Baphia punctulata* Harms subsp. *palmensis* Soladoye** | Strict-Endemic | Shrub or subshrub | NE | Mozambique | Kew Bull. 40: 361 (1985) | Pires de Lima 203 | Cabo Delgado, Palma | -10.7745 | 40.4737 | 1917 | X | Holotype (PO) |
| ***Bauhinia burrowsii* E.J.D.Schmidt** | Strict-Endemic | Shrub or subshrub | EN | Mozambique | Bothalia 42: 44 (2012) | Schmidt 5022 | Inhambane, near Mapinhane (East of Mapinhane) | -22.2231 | 34.9058 | 2010 |  | Holotype (PRE) |
| ***Bauhinia petersiana* Bolle** | Native | Shrub or subshrub | LC | Tanzania to Caprivi Strip | W.C.H.Peters, Naturw. Reise Mossambique 6(Bot., 1): 24 (1861) | Kirk s.n. | Zambézia, near Morrumbala, Rios de Sena | -17.3264 | 35.5858 | 1859 |  | Isotype (K) |
| *Bauhinia punctata* Bolle [=*Bauhinia galpinii* N.E.Br.] |  |  |  |  | W.C.H.Peters, Naturw. Reise Mossambique 6(Bot., 1): 23 (1861), nom. illeg. | Peters s.n. | Zambézia | -16.5883 | 36.8085 | Unknown |  | Isotype (K) |
| ***Berlinia orientalis* Brenan** | Near-Endemic | Tree | VU | Tanzania to Mozambique | Kew Bull. 17: 211 (1963) | Allen 95 | Cabo Delgado, Mouth of the M'salo [M'salu] River | -11.6704 | 40.4394 | 1911 |  | Holotype (K) |
| ***Brachystegia allenii* Burtt Davy & Hutch.** | Native | Tree | LC | Tanzania to S. Tropical Africa | Bull. Misc. Inform. Kew 1923: 156 (1923) | Allen 93 | Cabo Delgado, Mouth of the M'salo [M'salu] River | -11.6704 | 40.4394 | 1911 |  | Holotype (K) |
| ***Brachystegia oblonga* Sim** | Strict-Endemic | Tree | CR | Mozambique | Forest Fl. Port. E. Afr.: 49 (1909) | Sim 5574 | Zambezia, Quelimane, Maganja da Costa and around Arenga | -17.4056 | 37.4836 | 1908 |  | Not traced |
| ***Brachystegia torrei* Hoyle [=*Brachystegia tamarindoides* Welw. ex Benth. subsp. *torrei* (Hoyle) Chikuni]** | Native | Tree | NE^4^ | S. Tropical Africa | Kew Bull. 54: 159 (1999) | Torre 6141 | Manica, between Espungabera e Chibabava | -20.3604 | 33.1542 | 1943 |  | Holotype (FHO) |
| ***Calliandra xylocarpa* Sprague [=*Bussea xylocarpa* (Sprague) Sprague & Craib]** | Strict-Endemic | Tree | VU | Mozambique | Bull. Misc. Inform. Kew 1908: 290 (1908) | Kirk s.n. | Manica, Lupata | -16.6307 | 34.0264 | 1859 |  | Holotype (K) |
| ***Capassa violacea* Klotzsch [=*Philenoptera violacea* (Klotzsch) Schrire]** | Native | Tree | LC | S. Tropical & S. Africa | W.C.H.Peters, Naturw. Reise Mossambique 6(Bot., 1): 27 (1861) | Peters s.n. | Sofala, Vila de Sena | -17.4525 | 35.0325 | Unknown |  | Isotype (K) |
| ***Cassia abbreviata* Oliv.** | Native | Shrub or subshrub | LC | Tanzania to S. Tropical Africa | Fl. Trop. Afr. 2: 271 (1871) | Kirk s.n. | Manica, near Lupata | -16.6225 | 34.0265 | 1859 |  | Syntype (K) |
|  |  |  |  |  | Fl. Trop. Afr. 2: 271 (1871) | Kirk s.n. | Tete, near Tete | -16.0953 | 33.6419 | 1858 |  | Syntype (K) |
| ***Cassia afrofistula* Brenan var. *patentipila* Brenan [=*Cassia afrofistula* Brenan]** | Native | Shrub or subshrub | LC | Kenya to Mozambique, NE. Madagascar | Kew Bull. 13: 238 (1958) | Faulkner 343 | Zambézia, Lugela-Mocuba, Namagoa Estate | -16.5709 | 36.8575 | 1946 |  | Holotype (K) |
| *Cassia delagoensis* Harvey [=*Senna petersiana* (Bolle) Lock] |  |  |  |  | W.H.Harvey & auct. suc. (eds.), Fl. Cap. 2: 272 (1862) | Forbes s.n. | Maputo, Delagoa Bay | -26.0899 | 32.6398 | 1822 |  | Syntype (P) |
| ***Cassia paralias* Brenan [=*Chamaecrista paralias* (Brenan) Lock]** | Strict-Endemic | Perennial herb | LC | Mozambique | Kew Bull. 14: 187 (1960) | Gomes e Sousa 1691 | Inhambane, Dunas de Poméne, in sandy soils near the sea, 20 m | -22.9206 | 35.5825 | 1935 |  | Holotype (K) |
| ***Cassia zambesica* Oliv. [=*Chamaecrista zambesica* (Oliv.) Lock]** | Native | Perennial herb | NE | S. Somalia to S. Tropical Africa | Fl. Trop. Afr. 2: 280 (1871) | Kirk s.n. | Tete, between Tete and the sea coast, "Shamwara" | -14.9017 | 32.2136 | 1860 |  | Holotype (K) |
| ***Copaifera mopane* J.Kirk ex Benth. [=*Colophospermum mopane* (J.Kirk ex Benth.) J.Léonard]** | Native | Shrub or subshrub | LC | S. Tropical & S. Africa | Trans. Linn. Soc. London 25: 317 (1865) | Kirk s.n. | Manica, Lupata | -16.6307 | 34.0264 | 1860 |  | Syntype (K) |
|  |  |  |  |  | Trans. Linn. Soc. London 25: 317 (1865) | Kirk s.n. | Sofala, Chiramba | -16.9063 | 34.6469 | 1860 |  | Syntype (K) |
| ***Cordyla africana* Lour.** | Native | Tree | LC | Kenya to S. Africa | Fl. Cochinch.: 412 (1790) | Loureiro 500-1 | Mozambique |  |  | 1770 |  | Holotype (P) |
| ***Cracca bracteolata* var. *microfoliata* Pires de Lima [=*Tephrosia reptans* Baker var. *microfoliata* (P.Lima) Brummitt]** | Strict-Endemic | Annual or biennial herb | NE | Mozambique | Bol. Soc. Brot., sér. 2, 2: 137 (1924) | Pires de Lima 34 | Cabo Delgado, near Palma | -10.7640 | 40.4679 | 1916 |  | Holotype (PO) |
| *Cracca incana* (Roxb.) Pires de Lima [=*Tephrosia villosa* (L.) Pers. subsp. *ehrenbergiana* (Schweinf.) Brummitt] |  |  |  |  | Brotéria, Sér. Bot. 19: 124 (1921) | Pires de Lima 197 | Cabo Delgado, surroundings of Palma | -10.7758 | 40.4673 | 1917 |  | Holotype (PO) |
| ***Craibia filipes* Dunn [=*Craibia zimmermannii* (Harms) Dunn]** | Native | Shrub or subshrub | LC | Kenya to S. Africa | J. Bot. 49: 109 (1911) | Sheppard 305 | Sofala, 38 km from Beira, Zimbiti | -18.8880 | 35.5090 | 1909 |  | Holotype (K) |
| *Crotalaria arvensis* Klotzsch [=*Crotalaria podocarpa* DC.] |  |  |  |  | W.C.H.Peters, Naturw. Reise Mossambique 6(Bot., 1): 53 (1861) | Peters s.n. | Sofala, Vila de Sena | -17.4477 | 35.0353 | Unknown |  | Holotype (B)* |
| *Crotalaria ceciliae* Verdoorm [=*Crotalaria capensis* Jacq.] |  |  |  |  | Bothalia 2: 392 (1928) | Cecil 16 | Sofala, railway between Beira and Macequece | -19.2244 | 33.9570 | 1899 |  | Holotype (K) |
| ***Crotalaria dura* J.M.Wood & M.S.Evans subsp. *mozambica* Polhill** | Near-Endemic | Perennial herb | NE | Mozambique to KwaZulu-Natal | Crotalaria Africa & Madagascar: 128 (1982) | Torre 7364 | Maputo, between Moamba and Pessene | -25.6579 | 32.2999 | 1948 |  | Holotype (K) |
| *Crotalaria flexuosa* Baker [=*Crotalaria podocarpa* DC.] |  |  |  |  | D.Oliver & auct. suc. (eds.), Fl. Trop. Afr. 2: 29 (1871), nom. illeg. | Kirk s.n. | Tete | -16.1375 | 33.6067 | 1859 |  | Syntype (K) |
|  |  |  |  |  | D.Oliver & auct. suc. (eds.), Fl. Trop. Afr. 2: 29 (1871), nom. illeg. | Kirk s.n. | Tete, near Tete | -16.0953 | 33.6419 | 1860 |  | Syntype (K) |
| ***Crotalaria forbesii* Baker [=*Crotalaria virgulata* Klotzsch subsp. *forbesii* (Baker) Polhill]** | Native | Annual or biennial herb | NE | E. Tanzania to S. Tropical Africa | D.Oliver & auct. suc. (eds.), Fl. Trop. Afr. 2: 18 (1871) | Forbes s.n. | Sofala, probably Zambezi between Sena and coast | -17.4431 | 35.0505 | 1822 |  | Lectotype (K) |
| *Crotalaria gracilissima* Klotzsch [=*Crotalaria hyssopifolia* Klotzsch] |  |  |  |  | W.C.H.Peters, Naturw. Reise Mossambique 6(Bot., 1): 55 (1861) | Peters s.n. | Nampula, Cabaceira Peninsula | -14.9986 | 40.7593 | Unknown |  | Holotype (B)* |
| ***Crotalaria hyssopifolia* Klotzsch** | Native | Annual or biennial herb | NE | Tropical Africa | W.C.H.Peters, Naturw. Reise Mossambique 6(Bot., 1): 55 (1861) | Peters s.n. | Cabo Delgado, Quirimba Island | -12.4292 | 40.6045 | Unknown |  | Holotype (B)* |
| *Crotalaria junodiana* Baker f. [=*Crotalaria laburnoides* Klotzsch var. *laburnoides*] |  |  |  |  | J. Linn. Soc., Bot. 42: 341 (1914) | Junod 388 | Maputo, Delagoa Bay | -26.0899 | 32.6398 | 1893 |  | Holotype (Z) |
| ***Crotalaria lanceolata* E.Mey. subsp. *exigua* Polhill** | Near-Endemic | Annual or biennial herb | LC | S. Tropical Africa | Crotalaria Africa & Madagascar: 193 (1982) | Barbosa & Carvalho 2989 | Zambezia, between Mocuba and Milange, 46.7 km from Mocuba | -16.8218 | 36.6002 | 1949 |  | Isotype (LISC) |
| ***Crotalaria misella* Polhill** | Strict-Endemic | Annual or biennial herb | DD | Mozambique | Crotalaria Africa & Madagascar: 293 (1982) | Torre & Paiva 11873 | Cabo Delgado, Mueda, Macondes, 30 km from Chomba to Negomano, along the Matiu river | -11.5840 | 39.1438 | 1964 | X | Holotype (LISC) |
| ***Crotalaria mocubensis* Polhill** | Strict-Endemic | Annual or biennial herb | NE | Mozambique | Crotalaria Africa & Madagascar: 293 (1982) | Barbosa & Carvalho 2921 | Zambezia, Mocuba, Posto Agrícola | -16.8147 | 36.9944 | 1949 |  | Holotype (LISC) |
| ***Crotalaria monteiroi* Taub. ex Baker f. var. *monteiroi*** | Native | Perennial herb | NE | S. Mozambique to N. KwaZulu-Natal | J. Linn. Soc., Bot. 42: 387 (1914) | Monteiro 18 | Maputo, Delagoa Bay | -26.0899 | 32.6398 | 1876 |  | Lectotype (K) |
| *Crotalaria mossambicensis* Klotzsch [=*Crotalaria lanceolata* E.Mey. subsp. *lanceolata*] |  |  |  |  | W.C.H.Peters, Naturw. Reise Mossambique 6(Bot., 1): 60 (1861) | Peters s.n. | Mozambique |  |  | Unknown |  | Syntype (K) |
| ***Crotalaria namuliensis* Polhill & T.Harris** | Strict-Endemic | Annual or biennial herb | LC | Mozambique | Kew Bull. 66: 242 (2011) | Patel 7359 | Zambezia, Namuli Mountain, Muretha Plateau, 1916 m | -15.3911 | 37.0450 | 2007 |  | Holotype (K) |
| ***Crotalaria paraspartea* Polhill** | Strict-Endemic | Annual or biennial herb | EN | Mozambique | Crotalaria Africa & Madagascar: 221 (1982) | Torre & Paiva 11391 | Nampula, Namaita, between Nampula and Murrupula, region of Gafaria | -24.9096 | 34.2966 | 1964 |  | Holotype (LISC) |
|  |  |  |  |  | Crotalaria Africa & Madagascar: 221 (1982) | Torre & Paiva 11576 | Nampula, 27 km from Nampula to Muecate in the old road | -15.0876 | 39.4742 | 1964 |  | Paratype (LISC) |
| *Crotalaria pilifera* Klotzsch [=*Crotalaria podocarpa* DC.] |  |  |  |  | W.C.H.Peters, Naturw. Reise Mossambique 6(Bot., 1): 54 (1861) | Peters s.n. | Nampula, Mossuril and Cabaceira | -14.9572 | 40.7296 | Unknown |  | Syntype (B)* |
| ***Crotalaria preladoi* Baker f.** | Strict-Endemic | Annual or biennial herb | NE | Mozambique | J. Linn. Soc., Bot. 42: 367 (1914) | Prelado 38 | Nampula, Cabaceira Grande, on the beach sand | -15.0002 | 40.7595 | 1894 |  | Holotype (B)* [fragment of Holotype (BM)] |
| ***Crotalaria schlechteri* Baker f.** | Near-Endemic | Perennial herb | NE | S. Mozambique to Mpumalanga | J. Linn. Soc., Bot. 42: 365 (1914) | Schlechter 12037 | Maputo, Incanine | -25.6672 | 32.7242 | 1898 |  | Holotype (BM) |
| ***Crotalaria schliebenii* Polhill** | Near-Endemic | Annual or biennial herb | VU | SE. Tanzania to N. Mozambique | Kew Bull. 22: 328 (1968) | Balsinhas & Marrime 402 | Nampula, road from Nampula to Corrane, near the Colave river, ca. 10 km from Nampula | -15.1582 | 39.3337 | 1961 |  | Paratype (LISC) |
| *Crotalaria stewartii* Baker [=*Crotalaria polysperma* Kotschy] |  |  |  |  | D.Oliver & auct. suc. (eds.), Fl. Trop. Afr. 2: 32 (1871) | Stewart s.n. | Sofala, Shire & Shupanga, Zambeze | -18.0303 | 35.6148 | 1862 |  | Holotype (K) |
| ***Crotalaria torrei* Polhill** | Strict-Endemic | Shrub or subshrub | LC | Mozambique | Crotalaria Africa & Madagascar: 164 (1982) | Torre 5145 | Zambesia, Serra do Gurué, near Namúli peaks | -15.3767 | 37.0418 | 1943 |  | Holotype (LISC) |
| ***Crotalaria tunguensis* Pires de Lima [=*Crotalaria retusa* L. var. *tunguensis* (Pires de Lima) Polhill]** | Native | Perennial herb | NE | Somalia to Mozambique | Brotéria, Sér. Bot. 19: 120 (1921) | Pires de Lima 204 | Cabo Delgado, surroundings of Palma | -10.7758 | 40.4673 | 1917 |  | Holotype (PO) |
| ***Crotalaria virgulata* Klotzsch subsp. *virgulata*** | Native | Annual or biennial herb | NE | S. Tropical Africa | W.C.H.Peters, Naturw. Reise Mossambique 6(Bot., 1): 56 (1861) | Peters s.n. | Sofala, "Rios de Sena" | -17.4444 | 35.0448 | Unknown |  | Syntype (K) |
| ***Cynometra carvalhoi* Harms [=*Micklethwaitia carvalhoi* (Harms) G.P.Lewis & Schrire]** | Strict-Endemic | Tree | VU | Mozambique | Bot. Jahrb. Syst. 26: 261 (1899) | Augusto de Carvalho s.n. | Nampula, Mossuril to Cabeceira | -14.9536 | 40.7238 | 1884 |  | Holotype (COI) |
| ***Dalbergia arbutifolia* Baker** | Native | Shrub or subshrub | LC | DR Congo and Tanzania to S. Tropical Africa, Mayotte | D.Oliver & auct. suc. (eds.), Fl. Trop. Afr. 2: 232 (1871) | Kirk 22 | Tete, Near Manyerere, near Chicoa on the Zambezi. | -15.7389 | 32.3260 | 1860 |  | Holotype (K) |
| ***Dalbergia sambesiaca* Schinz** | Strict-Endemic | Tree | DD | Mozambique | Bull. Herb. Boissier, sér. 2, 2: 998 (1902) | Menyhart 843 | Zambezia, Boroma, Mutatatdzi [on the right side of the Zambesi-River, 16 km NW Tete] | -16.0786 | 33.4653 | 1891 |  | Holotype (WU) |
| *Desmodium afrum* var. *schlechteri* Schindl. [=*Grona afra* (E.Mey.) H.Ohashi & K.Ohashi] |  |  |  |  | Repert. Spec. Nov. Regni Veg. 23: 360 (1927) | Schlechter 11992 | Maputo, Delagoa Bay | -26.0899 | 32.6398 | 1898 |  | Syntype (K) |
| *Desmodium afrum* var. *schlechteri* Schindl. [=*Grona afra* (E.Mey.) H.Ohashi & K.Ohashi] |  |  |  |  | Repert. Spec. Nov. Regni Veg. 23: 360 (1927) | Schlechter 11705 | Maputo | -25.4486 | 32.3984 | 1897 |  | Isosyntype (K) |
| *Dialium mossambicense* Steyaert [=*Dialium holtzii* Harms] |  |  |  |  | Bull. Soc. Roy. Bot. Belgique 84: 39 (1951) | Gomes e Sousa 2302 | Niassa, Mecrussine forest | -12.5106 | 37.7515 | 1939 |  | Holotype (BR) |
| ***Dialium schlechteri* Harms** | Near-Endemic | Tree | LC | Mozambique to S. Africa | Bot. Jahrb. Syst. 26: 276 (1899) | Schlechter 11603 | Maputo | -25.4486 | 32.3984 | 1897 |  | Isotype (K) |
| ***Dichrostachys cinerea* (L.) Wight & Arn. subsp. *africana* Brenan & Brummitt** | Native | Shrub or subshrub | NE^4^ | Africa, Arabian Peninsula | Bol. Soc. Brot., sér. 2, 39: 77 (1965) | Gomes e Sousa 3466 | Maputo, Quinta do Umbeluzi | -26.0641 | 32.2167 | 1946 |  | Holotype (K) |
| ***Dichrostachys cinerea* (L.) Wight & Arn. var. *pubescens* Brenan & Brummitt** | Near-Endemic | Shrub or subshrub | NE^4^ | S. Tropical Africa to Eswatini | Bol. Soc. Brot., sér. 2, 39: 86 (1965) | Barbosa & Lemos 8149 | Gaza. Guijá, Aldeia de Barragem, left bank of Limpopo, forest gallery | -24.3947 | 32.8731 | 1957 |  | Holotype (K) |
| ***Dichrostachys forbesii* Benth. [=*Dichrostachys cinerea* (L.) Wight & Arn. subsp. *forbesii* (Benth.) Brenan & Brummitt]** | Native | Shrub or subshrub | NE^4^ | Kenya to KwaZulu-Natal | J. Bot. (Hooker) 4: 353 (1841) | Forbes s.n. | Maputo, Delagoa Bay | -26.0899 | 32.6398 | 1822 |  | Holotype (K) |
| *Dolichos brachypus* Harms [=*Neorautanenia mitis* (A.Rich.) Verdc.] |  |  |  |  | Bot. Jahrb. Syst. 26: 323 (1899) | Schlechter 11864 | Maputo, Komati Poort | -25.4644 | 31.9895 | 1897 |  | Holotype (P) |
| *Dolichos lupiniflorus* N.E.Br. [=*Dolichos kilimandscharicus* Taub. subsp. *kilimandscharicus*] |  |  |  |  | Bull. Misc. Inform. Kew 1906: 102 (1906) | Cecil 23 | Manica and Sofala, railway between Beira and Macequece | -19.2244 | 33.9570 | 1899 |  | Holotype (K) |
| ***Entada mossambicensis* Torre** | Strict-Endemic | Shrub or subshrub | VU | Mozambique | F.de A.Mendonça, Contr. Conhec. Fl. Mocamb. 2: 88 (1954) | Torre 4750 A | Nampula, surroundings of Nampula | -15.1031 | 39.3049 | 1942 |  | Holotype (LISC) |
|  |  |  |  |  | F.de A.Mendonça, Contr. Conhec. Fl. Mocamb. 2: 88 (1954) | Torre 1140 | Niassa, perto Nampula | -14.2907 | 37.3008 | 1936 |  | Paratype (LMA) |
| *Eriosema floribundum* Klotzsch [=Eriosema psoraleoides (Lam.) G.Don] |  |  |  |  | W.C.H.Peters, Naturw. Reise Mossambique 6(Bot., 1): 33 (1861), nom. illeg. | Peters s.n. | Sofala, vicinity of Rios de Sena from Quelimane to Tete | -17.4417 | 35.0473 | Unknown |  | Holotype (B)* |
| *Eriosema incanum* Klotzsch [=Eriosema psoraleoides (Lam.) G.Don] |  |  |  |  | W.C.H.Peters, Naturw. Reise Mossambique 6(Bot., 1): 35 (1861) | Peters s.n. | Cabo Delgado, Quirimba Island | -12.4292 | 40.6045 | Unknown |  | Holotype (B)* |
| *Eriosema macrophyllum* Klotzsch [=*Eriosema psoraleoides* (Lam.) G.Don] |  |  |  |  | W.C.H.Peters, Naturw. Reise Mossambique 6(Bot., 1): 34 (1861) | Peters s.n. | Nampula, mainland oposite Mozambique islands | -14.9776 | 40.6450 | Unknown |  | Holotype (B)* |
| ***Eriosema pauciflorum* Klotzsch** | Native | Perennial herb | LC | Tropical & S. Africa | W.C.H.Peters, Naturw. Reise Mossambique 6(Bot., 1): 31 (1861) | Peters s.n. | Sofala, Vila de Sena | -17.4548 | 35.0208 | Unknown |  | Holotype (B)* [drawing of Holotype (BM)] |
| *Eriosema sousae* Exell [=*Eriosema macrostipulum* Baker f.] |  |  |  |  | Bol. Soc. Brot., sér. 2, 12: 12 (1937) | Gomes e Sousa 1025 | Niassa, Unango, Catholic Mission | -12.8760 | 35.4101 | 1932 |  | Holotype (COI) |
| *Erythrina afra* var. *mossambicensis* Baker f. [=*Erythrina lysistemon* Hutch.] |  |  |  |  | J. Bot. 76: 238 (1938) | Torre 523, 523 | Niassa, Maniamba, region of Mepoche | -12.7664 | 34.9814 | 1934 |  | Holotype (LISC) |
| *Erythrina mossambicensis* Sim [=*Erythrina abyssinica* Lam.] |  |  |  |  | Forest Fl. Port. E. Afr.: 43 (1909) | Sim 5833 | Mozambique |  |  | 1908 |  | Not traced |
| ***Gorskia conjugata* Bolle [=*Guibourtia conjugata* (Bolle) J.Léonard]** | Native | Shrub or subshrub | LC | S. Tropical & S. Africa | W.C.H.Peters, Naturw. Reise Mossambique 6(Bot., 1): 15 (1861) | Peters s.n. | Sofala, vicinity of Sena and Tete | -16.7634 | 33.7010 | Unknown |  | Isotype (K) |
| ***Guibourtia sousae* J.Léonard** | Strict-Endemic | Tree | CR | Mozambique | Bull. Jard. Bot. État Bruxelles 20: 270 (1950) | Gomes e Sousa 1927 | Inhambane, Maueele, Panda District | -24.3463 | 34.1331 | 1936 | X | Holotype (K) |
| ***Icuria dunensis* Wieringa** | Strict-Endemic | Tree | EN | Mozambique | Wageningen Agric. Univ. Pap. 99(4): 242 (1999) | Johnson & Avis 694 | Nampula, Moma regions, in SW of BHP concession, 68 km SW Angoche, 5 m | -16.6779 | 39.4767 | 1998 |  | Holotype (WAG) |
| ***Indigofera concinna* Baker** | Near-Endemic | Annual or biennial herb | NE | Tanzania to Mozambique | D.Oliver & auct. suc. (eds.), Fl. Trop. Afr. 2: 80 (1871) | Kirk s.n. | Cabo Delgado, Rovuma river | -10.9418 | 39.7207 | 1861 |  | Holotype (K) |
| ***Indigofera delagoaensis* Baker f. ex J.B.Gillett** | Native | Perennial herb | NE | S. Tropical & S. Africa | Kew Bull., Addit. Ser. 1: 67 (1958) | Rogers 21372 | Maputo | -25.4486 | 32.3984 | 1915 |  | Isoparatype (GRA) |
|  |  |  |  |  | Kew Bull., Addit. Ser. 1: 67 (1958) | Schlechter 11532 | Maputo | -25.4486 | 32.3984 | 1897 |  | Holotype (K) |
| ***Indigofera dyeri* Britten var. *parviflora* J.B.Gillett** | Native? | Annual or biennial herb | NE | S. Tropical Africa | Kew Bull. 24: 493 (1970) | Lemos & Macuacua 127 | Sofala, Chemba, Estação Experimental de C.I.C.A. | -17.1639 | 34.8893 | 1960 |  | Isotype (K) |
| *Indigofera dyeri* var. *congesta* J.B. Gillett. [=*Indigofera pseudodyeri* Schrire] |  |  |  |  | Kew Bull. 24: 493 (1970) | Faulkner 74 | Zambézia, Lugela-Mocuba, Namagoa Estate | -16.5709 | 36.8575 | 1946 |  | Holotype (K) |
| ***Indigofera emarginella* Steud. ex A.Rich. var. *marrupaensis* Schrire** | Strict-Endemic | Shrub or subshrub | NE | Mozambique | Kew Bull. 53: 661 (1998) | Nuvunga 434 | Niassa, Marrupa | -13.1841 | 37.4977 | 1981 | X | Holotype (K) |
| ***Indigofera erythrogramma* Welw. ex Baker subsp. *nampulensis* Schrire** | Near-Endemic | Annual or biennial herb | NE | Malawi to Mozambique | Fl. Zambes. 3(4): 141 (2012) | Balsinhas & Marrime 443 | Nampula, Malema, Mutuáli, Estação experimental de CICA | -14.8756 | 37.0076 | 1961 |  | Isotype (BR) |
| ***Indigofera faulknerae* J.B.Gillett** | Native | Annual or biennial herb | NE | Malawi to Mozambique | Kew Bull., Addit. Ser. 1: 23 (1958) | Faulkner 227 | Zambezia, Quelimane District. Road to Moebede, Lugela district | -16.5824 | 36.7499 | 1948 |  | Isotype (K) |
| ***Indigofera fulgens* Baker subsp. *fulgens*** | Native | Shrub or subshrub | NE | Tanzania to Northern Prov. | D.Oliver & auct. suc. (eds.), Fl. Trop. Afr. 2: 101 (1871) | Kirk s.n. | Cabo Delgado, banks of the Rovuma river | -10.9408 | 39.7179 | 1861 |  | Holotype (K) |
| ***Indigofera gobensis* Schrire** | Strict-Endemic | Perennial herb | CR | Mozambique | Fl. Zambes. 3(4): 93 (2012) | Balsinhas 522 | Maputo, Namaacha, near Goba-Fronteira | -26.2556 | 32.0911 | 1961 |  | Holotype (K) |
| ***Indigofera hedyantha* Eckl. & Zeyh. subsp. *robusta* Schrire** | Native | Perennial herb | NE | Mozambique to Cape Prov. | Fl. Zambes. 3(4): 98 (2012) | Lemos & Balsinhas 188 | Maputo, Namaacha District, Goba-Fronteira, near war memorial | -26.2520 | 32.0885 | 1961 |  | Holotype (LISC) |
| ***Indigofera inhambanensis* Klotzsch** | Native | Perennial herb | NE | S. Tropical Africa | W.C.H.Peters, Naturw. Reise Mossambique 6(Bot., 1): 48 (1861) | Forbes s.n. | Maputo, Delagoa Bay | -26.0899 | 32.6398 | 1822 |  | Holotype (K) |
| ***Indigofera kirkii* Oliv.** | Native | Shrub or subshrub | NE | Kenya to Mozambique, NW. Madagascar | Hooker's Icon. Pl. 15: t. 1416 (1883) | Kuntze s.n. | Mozambique |  |  | 1894 |  | Neotype (K) |
| ***Indigofera kuntzei* Harms** | Native | Perennial herb | DD | Tanzania to S. Tropical Africa | C.E.O.Kuntze, Revis. Gen. Pl. 3(2): 51 (1898) | Kuntze s.n. | Mozambique |  |  | 1894 |  | Isotype (K) |
| ***Indigofera laxeracemosa* Baker f.** | Native | Annual or biennial herb | NE | Tanzania to KwaZulu-Natal | J. Bot. 41: 241 (1903) | Kirk s.n. | Sofala, Kongone mouth of Zambesi | -19.8433 | 34.7652 | 1860 |  | Isotype (K) |
| ***Indigofera lupatana* Baker f.** | Native | Shrub or subshrub | NE | Ethiopia to Northern Prov. | Legum. Trop. Africa: 154 (1926) | Kirk s.n. | Manica, on Zambesi | -16.6611 | 33.9992 | 1860 |  | Holotype (K) |
| ***Indigofera mendoncae* J.B.Gillett** | Strict-Endemic | Perennial herb | DD | Mozambique | Kew Bull., Addit. Ser. 1: 46 (1958) | Mendonca 3315 | Inhambane, between Quissico and Chicomo | -24.6536 | 34.5681 | 1944 |  | Holotype (K) |
| *Indigofera moniliformis* Baker f. [=*Indigofera ormocarpoides* Baker ] |  |  |  |  | J. Bot. 41: 323 (1903) | Kirk s.n. | Manica, between Lupata and Tete | -16.6210 | 34.0262 | 1859 |  | Syntype (K) |
| *Indigofera mossambicensis* Baker f. [=*Indigofera concinna* Baker] |  |  |  |  | J. Bot. 70: 253 (1932) | Schlechter s.n. | Nampula, Mozambique Island | -15.0329 | 40.7333 | 1895 |  | Holotype (B)* [fragment of Holotype (BM)] |
| *Indigofera multijuga* Baker [*Indigofera dendroides* Jacq.] |  |  |  |  | D.Oliver & auct. suc. (eds.), Fl. Trop. Afr. 2: 82 (1871) | Kirk s.n. | Sofala, Chupanga, on Zambesi | -18.0349 | 35.6277 | 1862 |  | Holotype (K) |
| ***Indigofera namuliensis* Schrire** | Strict-Endemic | Annual or biennial herb | DD | Mozambique | Fl. Zambes. 3(4): 69 (2012) | Patel 7413 | Zambezia, Namuli Mountain, below Ukatini Forest below peak of Mt. Namuli, 1400-1500 m | -15.3744 | 37.0669 | 2007 |  | Holotype (K) |
| ***Indigofera oligophylla* Klotzsch** | Native | Perennial herb | LC | Tanzania to Mozambique | W.C.H.Peters, Naturw. Reise Mossambique 6(Bot., 1): 49 (1861) | Gomes e Sousa 3512 | Nampula, Goa Island | -15.0528 | 40.7842 | 1947 |  | Neotype (K) |
| ***Indigofera podophylla* Harv.** | Near-Endemic | Perennial herb | NE | Mozambique to KwaZulu-Natal | W.H.Harvey & auct. suc. (eds.), Fl. Cap. 2: 168 (1862) | Forbes s.n. | Maputo, Delagoa Bay | -26.0899 | 32.6398 | 1822 |  | Isotype (K) |
| ***Indigofera pseudomoniliformis* Schrire** | Strict-Endemic | Shrub or subshrub | VU | Mozambique | Kew Bull. 53: 662 (1998) | Torre & Correia 17399 | Nampula, Mogovolas, 21 km on the road to luluti, Nhopera mount | -15.6810 | 39.2029 | 1968 |  | Holotype (LISC) |
| ***Indigofera rostrata* Bolus subsp. *namaachensis* Schrire** | Native | Perennial herb | NE | Mozambique to S. Africa | Fl. Zambes. 3(4): 97 (2012) | Exell & Mendonca & Wild 496 | Maputo, Lebombo Mts, Mt Mponduine, near Namaacha | -25.9476 | 31.9777 | 1955 |  | Holotype (BM) |
| *Indigofera sousae* M.A.Exell [=*Indigofera atriceps* Hook.f. subsp. *glandulosissima* (R.E.Fr.) J.B.Gillett] |  |  |  |  | Bol. Soc. Brot., sér. 2, 12: 8 (1937) | Gomes e Sousa 1284 | Niassa, Massangulo | -13.9001 | 35.4210 | 1933 |  | Isotype (K) |
| *Indigofera tettensis* Klotzsch [=*Indigofera schimperi* Jaub. & Spach var. *schimperi*] |  |  |  |  | W.C.H.Peters, Naturw. Reise Mossambique 6(Bot., 1): 51 (1861) | Peters s.n. | Tete | -16.0814 | 33.5699 | Unknown |  | Holotype (B)* |
| ***Indigofera torrei* J.B.Gillett** | Strict-Endemic | Perennial herb | VU | Mozambique | Kew Bull., Addit. Ser. 1: 58 (1958) | Torre 7837 | Gaza, Chamusca region, Canicado. Limpopo district, from Camicado on Saute road. | -24.4720 | 33.0135 | 1948 |  | Holotype (K) |
| *Indigofera torulosa* Baker [=*Indigofera ormocarpoides* Baker ] |  |  |  |  | D.Oliver & auct. suc. (eds.), Fl. Trop. Afr. 2: 91 (1871), nom. illeg. | Kirk s.n. | Niassa, Rovuma river, 16 miles up | -11.4244 | 38.4733 | 1861 |  | Syntype (K) |
| *Indigofera torulosa* Baker [=*Indigofera ormocarpoides* Baker ] |  |  |  |  | D.Oliver & auct. suc. (eds.), Fl. Trop. Afr. 2: 91 (1871), nom. illeg. | Kirk s.n. | Manica, Lupata | -16.6307 | 34.0264 | 1860 |  | Syntype (K) |
| ***Lonchocarpus capassa* Rolfe** | Native | Tree | NE | Tanzania to S. Africa | F.Oates, Matabele Land, ed. 2: 897 (1889) | Peters s.n. | Sofala, Vila de Sena | -17.4525 | 35.0325 | Unknown |  | Isotype (K) |
| *Lonchocarpus menyharthii* Schinz [=*Philenoptera bussei* (Harms) Schrire] |  |  |  |  | Bull. Herb. Boissier ser. 2, 2: 998 (5 Dec. 1902) | Menyhart 854 | Tete, Boroma | -16.0462 | 33.4448 | 1892 |  | Holotype (Z) |
| *Lonchocarpus mossambicensis* Sim [=*Millettia stuhlmannii* Taub.] |  |  |  |  | Forest Fl. Port. E. Afr.: 45 (1909) | Sim 5382 | Mozambique |  |  | 1908 |  | Holotype (PRE) |
| ***Lotus namulensis* Brand** | Native | Perennial herb | NE | S. Tropical Afroca to Northern Prov. | Bot. Jahrb. Syst. 25: 213 (1898) | Last s.n. | Zambézia, Namuli, Makua country | -15.3578 | 37.0606 | 1887 |  | Isotype (K) |
| ***Lotus wildii* J.B.Gillett** | Near-Endemic | Perennial herb | NE | Zimbabwe to Mozambique | Kew Bull. 13: 370 (1958 publ. 1959) | Torre 5944 | Sofala, Gorongosa, Cume da serra da Gorongosa (Gogôgo) | -18.4205 | 34.1055 | 1943 |  | Paratype (LISC) |
| ***Macrotyloma decipiens* Verdc.** | Strict-Endemic | Perennial herb | DD | Mozambique | Hooker's Icon. Pl. 38: t. 3778 (1982) | Pedro & Pedrogao 3130 | Nampula, Mossuril, Lumbo, 4.5 km (7 km on EA sheet) on road to Nampula | -15.0106 | 40.6643 | 1948 | X | Holotype (K) |
| ***Microcharis latifolia* Benth.** | Native | Annual or biennial herb | NE | Kenya to Northern Prov. | Trans. Linn. Soc. London 25: 298 (1865) | Kirk 41 | Zambézia, near mouth of Zambesi; right bank of Luabo; | -18.3975 | 36.0994 | 1856 |  | Holotype (K) |
| ***Millettia mossambicensis J.B.Gillett*** | Strict-Endemic | Tree | LC | Mozambique | Kew Bull. 15: 23 (1961) | Chase 5078 | Sofala, Gorongosa Game Reserve, woodland, 90 m | -18.7869 | 34.4941 | 1953 |  | Holotype (K) |
| ***Millettia stuhlmannii* Taub.** | Native | Tree | NE | Tanzania to Northern Prov. | H.G.A.Engler, Pflanzenw. Ost-Afrikas, C: 212 (1895) | Stuhlmann 868 | Zambézia," Pugaruni", 10 hours North of Quelimane | -15.5268 | 36.9844 | 1889 |  | Syntype (HBG) |
| ***Millettia stuhlmannii* Taub.** |  |  |  |  | H.G.A.Engler, Pflanzenw. Ost-Afrikas, C: 212 (1895) | Stuhlmann 856 | Mozambique |  |  | 1889 |  | Syntype (HBG) |
| ***Millettia usaramensis* Taub. subsp. *australis* J.B.Gillett** | Native | Shrub or subshrub | NE^4^ |  | Kew Bull. 15: 30 (1961) | Swynnerton 1425 | Sofala, Chironda, lower Búzi | -19.8952 | 34.6613 | 1906 |  | Holotype (K) |
| ***Mimosa mossambicensis* Brenan** | Strict-Endemic | Shrub or subshrub | LC | Mozambique | W.C.H.Peters, Naturw. Reise Mossambique 6(Bot., 1): 8 (1861), nom. illeg. | Peters s.n. | Sofala, Zambesi, Sena and Tette | -17.4274 | 35.0464 | Unknown |  | Isotype (K) |
| ***Mucuna coriacea* Baker susp. *coriacea*** | Native | Woody climber | NE | Tanzania to S. Tropical Africa | D.Oliver & auct. suc. (eds.), Fl. Trop. Afr. 2: 187 (1871) | Kirk s.n. | Zambézia, Chupanga | -18.0189 | 35.6153 | 1862 |  | Lectotype (K) |
| *Mucuna quadrialata* Baker [=*Mucuna gigantea* (Willd.) DC. subsp. *gigantea*] |  |  |  |  | D.Oliver & auct. suc. (eds.), Fl. Trop. Afr. 2: 186 (1871) | Kirk s.n. | Zambézia, Luabo River, Muselo | -18.3826 | 36.1204 | 1858 |  | Syntype (K) |
| *Mucuna rhynchosioides* Taub. [=*Mucuna coriacea* Baker susp. *coriacea*] |  |  |  |  | Bot. Jahrb. Syst. 23: 194 (1896) | Augusto de Carvalho s.n. | Mozambique, Zambezi river |  |  | 1884 |  | Holotype (B)* |
| *Mucuna rhynchosioides* Taub. [=*Mucuna coriacea* Baker susp. *coriacea*] |  |  |  |  | Bot. Jahrb. Syst. 23: 194 (1896); | Koning 8908 | Inhambane, Massinga, on the road to Rio das Pedras, 4 km walk | -23.2934 | 35.3791 | 1981 |  | Neotype (MO) |
| *Ormocarpum setosum* Burtt Davy [=*Ormocarpum trichocarpum* (Taub.) Engl.] |  |  |  |  | Man. Pl. Transvaal 2: 425 (1932) | Johnson 221 | Manica, Chimanimani Mts., Moribane | -19.7666 | 33.3014 | 1907 |  | Holotype (K) |
| ***Ormocarpum zambesianum V*erdc.** | Native | Shrub or subshrub | NE | Zimbabwe to Mozambique | Fl. Zambes. 3(6): 57 (2000) | Macedo 5496 | Tete, Zumbo, on the way to the mountains, north of the village | -15.5964 | 30.4485 | 1974 |  | Holotype (LISC) |
| ***Phaseolus schlechteri* Harms [=*Decorsea schlechteri* (Harms) Verdc.]** | Native | Perennial herb | NE | Tanzania to S. Africa | Bot. Jahrb. Syst. 30: 91 (1901) | Schlechter 11826 | Maputo, Ressano Garcia | -25.4429 | 31.9923 | 1897 |  | Isotype (GRA) |
| *Piptadenia lujae* De Wild. [=*Newtonia buchananii* (Baker) G.C.C. Gilbert & Boutique] |  |  |  |  | Ann. Soc. Sci. Bruxelles 44(1): 215 (1925) | Luja 446 | Zambézia, Morrumbala | -17.3345 | 35.5856 | 1901 |  | Holotype (BR) |
| ***Piptadenia schlechteri* Harms [=*Adenopodia schlechteri* (Harms) Brenan]** | Strict-Endemic | Woody climber | VU | Mozambique | Bot. Jahrb. Syst. 26: 260 (1899) | Schlechter 11700 | Maputo, Maputo | -25.8920 | 32.6327 | 1897 |  | Isotype (K) |
| ***Pterocarpus brenanii* L.Barb. & Torre** | Native | Tree | LC | S. Tropical Africa | Garcia de Orta 5: 12 (1957) | Andrada 1621 | Tete, Mutarara, road to Ancuaze | -17.3209 | 35.0346 | 1949 |  | Paratype (LISC) |
|  |  |  |  |  | Garcia de Orta 5: 12 (1957) | Andrada 1050 | Sofala, Inhaminga, near Bué Maria | -18.6880 | 34.0773 | 1948 |  | Paratype (LISC) |
|  |  |  |  |  | Garcia de Orta 5: 12 (1957) | Barbosa & Carvalho 3419 | Tete, between Chitengo and Tete, 41,9 km of Chitima | -15.8832 | 33.0154 | 1949 |  | Paratype (LMA) |
|  |  |  |  |  | Garcia de Orta 5: 12 (1957) | Barbosa & Carvalho 3235 | Tete, Moatize, between Nicungas and the junction, 5 km from Nicungas | -16.0971 | 33.6360 | 1949 |  | Paratype (LISC) |
|  |  |  |  |  | Garcia de Orta 5: 12 (1957) | Simao 1052 | Sofala, Gorongosa | -18.6954 | 34.4929 | 1946 |  | Paratype (LISC) |
|  |  |  |  |  | Garcia de Orta 5: 12 (1957) | Simao 608 | Sofala, Cheringoma, Inhamitanga | -18.2164 | 35.1589 | 1945 |  | Paratype (LISC) |
|  |  |  |  |  | Garcia de Orta 5: 12 (1957) | Torre 6015 | Tete, entre Tete e Boroma | -16.8304 | 34.8764 | 1943 |  | Holotype (LISC) |
|  |  |  |  |  | Garcia de Orta 5: 12 (1957) | Torre 3103 | Sofala, between Chemba and Marínguè | -17.4872 | 34.5614 | 1941 |  | Paratype (LISC) |
| ***Rhynchosia chimanimaniensis* Verdc.** | Near-Endemic | Perennial herb | EN | Zimbabwe to Mozambique | Kew Bull. 55: 144 (2000) | Wild 2926 | Manica, Chimanimani Mts. | -19.8428 | 33.1055 | 1949 |  | Holotype (K) |
| ***Rhynchosia clivorum* S.Moore subsp. *gurueensis* Verdc.** | Strict-Endemic | Perennial herb | DD | Mozambique | Fl. Zambes. 3(5): 179 (2001) | Mendonca 2143 | Zambezia, Gurué, Serra Namuli, Serra do Gurué, on summit | -15.3742 | 37.0406 | 1944 | X | Holotype (LISC) |
| *Rhynchosia melanosperma* Klotzsch [=*Rhynchosia caribaea* (Jacq.) DC.] |  |  |  |  | W.C.H.Peters, Naturw. Reise Mossambique 6(Bot., 1): 30 (1861) | Peters s.n. | Sofala, Vila de Sena | -17.4525 | 35.0325 | 1846 |  | Isotype (K) |
| ***Rhynchosia torrei* Verdc.** | Strict-Endemic | Shrub or subshrub | LC | Mozambique | Fl. Zambes. 3(5): 178 (2001) | Torre 3556 | Zambezia, Gúruè, nas encostas dos montes | -15.4292 | 37.0333 | 1941 |  | Holotype (LISC) |
| ***Rhynchosia velutina* Wight & Arn. var. *discolor* (Bak.) Verdc.** | Native | Perennial climbing herb | VU | Somalia to Mozambique, Comoros, Madagascar, India, Sri Lanka | Kew Bull. 25: 92 (1971) | Peters s.n. | Inhambane | -23.8662 | 35.4010 | Unknown |  | Holotype (B)* |
| *Saldania aconthocarpa* Taub. Engl. [=*Ormocarpum trichocarpum* (Taub.) Engl.] |  |  |  |  | Forest Fl. Port. E. Afr.: 42 (1909) | Sim 6222 | Maputo, frequent in the Umbelúzi Valley and thence to the Lebombo Range | -26.0960 | 32.3437 | 1908 |  | Holotype (NU) |
| ***Schotia capitata* Bolle** | Native | Shrub or subshrub | LC | S. Tropical & S. Africa | W.C.H.Peters, Naturw. Reise Mossambique 6(Bot., 1): 18 (1861) | Balsinhas 204 | Maputo, Goba, proximidades do rio Maivavo. | -26.2000 | 32.1227 | 1960 |  | Neotype (K) |
| *Schotia tamarindifolia* var. *forbesiana* Baill. [=*Schotia capitata* Bolle] |  |  |  |  | Adansonia 6: 197 (1866) | Forbes 32 | Maputo, Delagoa Bay | -26.0899 | 32.6398 | 1822 |  | Holotype (P) |
| ***Scorodophloeus torrei* Lock** | Strict-Endemic | Shrub or subshrub | EN | Mozambique | Kew Bull. 61: 257 (2006) | Patacas s.n. | Sofala, Angoche [Antonio Enes], Farol de Sangage | -16.0065 | 40.1194 | 1970 |  | Holotype (LISC) |
|  |  |  |  |  | Kew Bull. 61: 257 (2006) | Torre & Correia 14543 | Zambezia, Maganja da Costa, Gobene Forest, at km 50 from Maganja Village, road to the beach | -17.4455 | 37.5286 | 1966 |  | Paratype (LISC) |
|  |  |  |  |  | Kew Bull. 61: 257 (2006) | Torre & Correia 17320 | Sofala, Angoche [Antonio Enes], Farol de Sangage | -16.0065 | 40.1194 | 1968 |  | Paratype (LISC) |
|  |  |  |  |  | Kew Bull. 61: 257 (2006) | Torre & Correia 14585 | Zambezia, Maganja da Costa, Gobene Forest, at km 40 from Maganja Village, road to the beach | -17.4455 | 37.5286 | 1966 |  | Paratype (LISC) |
|  |  |  |  |  | Kew Bull. 61: 257 (2006) | Torre & Paiva 9476 | Nampula, Memba, granite hill near Memba, ca. 300 m from the sea | -14.1858 | 40.5258 | 1963 |  | Paratype (LISC) |
|  |  |  |  |  | Kew Bull. 61: 257 (2006) | Torre & Paiva 17006 | Zambezia, Maganja da Costa, Gobene Forest, at km 35 from Maganja Village | -17.4455 | 37.5286 | 1968 |  | Paratype (LISC) |
|  |  |  |  |  | Kew Bull. 61: 257 (2006) | Torre & Paiva 17056 | Zambezia, Maganja da Costa, Gobene Forest, near Razaga beach, 40 km from Maganja Village | -17.5865 | 37.4864 | 1968 |  | Paratype (LISC) |
| ***Sesbania greenwayi* J.B.Gillett** | Native | Annual or biennial herb | NE | Somalia to S. Tropical Africa | Kew Bull. 17: 138 (1963) | Chase 2717 | Zambézia, Boroma, Zambezi River, Msusa | -16.0474 | 33.4449 | 1950 |  | Paratype (LISC) |
|  |  |  |  |  | Kew Bull. 17: 138 (1963) | Lemos & Macuacua 137 | Sofala, Chemba, on the road to Tambara | -17.0550 | 34.8320 | 1960 |  | Paratype (LISC) |
| *Sesbania kirkii* E.Phillips & Hutch. [=*Sesbania tetraptera* Hochst. ex Baker subsp. *tetraptera*] |  |  |  |  | Bothalia 1: 54 (1921) | Kirk s.n. | Sofala, near Sena | -17.4525 | 35.0325 | 1859 |  | Syntype (K) |
| ***Sesbania mossambicensis*Klotzsch** | Native | Annual or biennial herb | NE | S. Tropical Africa | W.C.H.Peters, Naturw. Reise Mossambique 6(Bot., 1): 45 (1861) | Peters s.n. | Mozambique |  |  | Unknown |  | Isosyntype (K) |
| ***Sophora inhambanensis* Klotzsch** | Native | Shrub or subshrub | LC | Kenya to KwaZulu-Natal, Madagascar | W.C.H.Peters, Naturw. Reise Mossambique 6(Bot., 1): 26 (1861) | Kirk s.n. | Zambézia, Zambesi mouth, coast | -18.8538 | 36.3138 | 1862 |  | Neotype (K) |
|  |  |  |  |  | W.C.H.Peters, Naturw. Reise Mossambique 6(Bot., 1): 26 (1861) | Peters s.n. | Inhambane | -22.8004 | 34.5142 | Unknown |  | Syntype (B)* |
|  |  |  |  |  | W.C.H.Peters, Naturw. Reise Mossambique 6(Bot., 1): 26 (1861) | Peters s.n. | Maputo, Delagoa Bay | -26.0899 | 32.6398 | Unknown |  | Syntype (B)* |
| *Tephrosia aequilata* Baker subsp. *namuliana* Brummitt [=*Tephrosia aequilata* Baker] |  |  |  |  | Bol. Soc. Brot., sér. 2, 41: 356 (1968) | Leach & Schelpe 11471 | Zambézia, Namuli Peaks, West face | -15.1317 | 37.2497 | 1962 |  | Holotype (K) |
| *Tephrosia aequilata* Baker subsp. *gorongosana* Brummitt [=*Tephrosia aequilata* Baker] |  |  |  |  | Bol. Soc. Brot., sér. 2, 41: 357 (1968) | Torre & Paiva 12305 | Sofala, Gorongosa, Serra da Gorongosa, at the waterfalls | -19.0181 | 34.2862 | 1964 |  | Holotype (LISC) |
| ***Tephrosia argyrotricha* Harms** | Native | Annual or biennial herb | NE | Burundi to Mozambique | C.E.O.Kuntze, Revis. Gen. Pl. 3(2): 57 (1898) | Kuntze s.n. | Mozambique |  |  | 1894 |  | Isotype (NY) |
| *Tephrosia carvalhoi* Taub. [=*Tephrosia reptans* Baker var. *reptans*] |  |  |  |  | Bot. Jahrb. Syst. 23: 183 (1896) | Augusto de Carvalho s.n. | Sofala, Gorongosa | -18.6954 | 34.4929 | 1884 |  | Isotype (COI) |
| ***Tephrosia crotalarioides* Klotzsch [=*Indigofera crotalarioides* (Klotzsch) Baker]** | Strict-Endemic | Annual or biennial herb | NE | Mozambique | W.C.H.Peters, Naturw. Reise Mossambique 6(Bot., 1): 45 (1861) | Peters s.n. | Zambezia, Zambesi land |  |  | Unknown |  | Holotype (B)* |
| *Tephrosia delagoensis* H.M.L.Forbes [=*Tephrosia purpurea* (L.) Pers. var. *delagoensis* (H.M.L.Forbes) Brummitt] |  |  |  |  | Bothalia 4: 968 (1948) | Schlechter 11521 | Maputo, *in arenosis* | -25.9089 | 32.6544 | 1897 |  | Holotype (PRE) |
| *Tephrosia ehrenbergiana* Schweinf. [=*Tephrosia villosa* (L.) Pers. subsp. *ehrenbergiana* (Schweinf.) Brummitt] |  |  |  |  | Beitr. Fl. Aethiop.: 18 (1867) | Peter s.n. | Mozambique |  |  | Unknown |  | Isosyntype (K) |
| ***Tephrosia faulknerae* Brummitt** | Strict-Endemic | Shrub or subshrub | EN | Mozambique | Bol. Soc. Brot., sér. 2, 41: 282 (1968) | Faulkner 404 | Zambezia, Quelimane District, Namagoa, Mocuba and Moebeda road, Lugela | -16.5864 | 36.8717 | 1949 |  | Isotype (LISC) |
|  |  |  |  |  | Bol. Soc. Brot., sér. 2, 41: 282 (1968) | Torre 1192 | Nampula, Arredores de Nampula | -15.0864 | 39.1985 | 1937 |  | Paratype (LISC) |
| ***Tephrosia forbesii* Baker subsp. *inhacensis* Brummitt** | Strict-Endemic | Perennial herb | VU | Mozambique | Bol. Soc. Brot., sér. 2, 41: 277 (1968) | Mogg 29869 | Maputo, Inhaca Island, w. coast ridge grassland, 25 miles E. of Maputo | -26.0424 | 32.9057 | 1962 |  | Holotype (K) |
| ***Tephrosia gobensis* Brummitt** | Near-Endemic | Shrub or subshrub | VU | Mozambique to Eswatini | Bol. Soc. Brot., sér. 2, 41: 368 (1968) | Esteves de Sousa 132 | Maputo, near the fountain "Fonte-dos-Cibombos", 9.5 kilometres from Goba. | -26.1501 | 32.1102 | 1945 |  | Holotype (LISC) |
| *Tephrosia incarnata* Brummitt [=*Tephrosia glomeruliflora* Meisn. subsp. *meisneri* (Hutch. & Burtt Davy) Schrire] |  |  |  |  | Bol. Soc. Brot., sér. 2, 41: 370 (1968) | Barbosa & Lemos 7534 | Maputo, Namaacha, near the Canada Dry company. | -25.9393 | 31.9909 | 1957 |  | Paratype (LISC) |
| *Tephrosia indigofera* Bertol. [=Tephrosia purpurea (L.) Pers. subsp. purpurea] |  |  |  |  | Misc. Bot. 19: 9, t. 5 (1858) | Fornasini s.n. | Inhambane | -23.8681 | 35.3993 | 1839-1868 |  | Holotype (BOLO) [photograph of Holotype (K)] |
| *Tephrosia junodii* De Wild [=*Tephrosia forbesii* Baker subsp. *forbesii*] |  |  |  |  | Ann. Mus. Congo Belge, Bot., sér. 5, 1: 261 (1906) | Junod 168 | Maputo, Delagoa Bay | -26.0899 | 32.6398 | 1890 |  | Holotype (BR) |
| *Tephrosia kirkii* Baker [=*Tephrosia reptans* Baker var. *reptans*] |  |  |  |  | D.Oliver & auct. suc. (eds.), Fl. Trop. Afr. 2: 115 (1871) | Kirk s.n. | Zambezia, Luawe River, 'Livingston's Zambesi Expedition' | -18.4031 | 36.0994 | 1861 |  | Holotype (K) |
| *Tephrosia longipes* Meisn. var. *icosisperma* Brummitt [=*Tephrosia longipes* Meisn. var. *longipes*] |  |  |  |  | Bol. Soc. Brot., sér. 2, 41: 313 (1968) | Barbosa 1411 | Manica, Manica, Mavita, near Chinacata village | -19.5204 | 33.1509 | 1948 |  | Paratype (LISC) |
|  |  |  |  |  | Bol. Soc. Brot., sér. 2, 41: 313 (1968) | Barbosa 49 | Maputo, Goba | -26.2025 | 32.1321 | 1947 |  | Paratype (LISC) |
|  |  |  |  |  | Bol. Soc. Brot., sér. 2, 41: 313 (1968) | Barbosa 1443 | Manica, Mavita, Rutanda | -19.5410 | 33.0296 | 1948 |  | Paratype (LISC) |
|  |  |  |  |  | Bol. Soc. Brot., sér. 2, 41: 313 (1968) | Barbosa 1074 | Manica, Chimoio, base of Garuzo Mountain | -18.9400 | 33.0698 | 1948 |  | Paratype (LISC) |
|  |  |  |  |  | Bol. Soc. Brot., sér. 2, 41: 313 (1968) | Barbosa 7879 | Maputo, Vila Luisa, Campo Experimental de Estudos Arborícolas | -25.7373 | 32.6747 | 1957 |  | Paratype (LISC) |
|  |  |  |  |  | Bol. Soc. Brot., sér. 2, 41: 313 (1968) | Barbosa 38 | Maputo, Goba | -26.2027 | 32.1315 | 1947 |  | Paratype (LISC) |
|  |  |  |  |  | Bol. Soc. Brot., sér. 2, 41: 313 (1968) | Barbosa 965 | Manica, Manica, between Rotanda and Mavita | -19.5602 | 33.0593 | 1948 |  | Paratype (LISC) |
|  |  |  |  |  | Bol. Soc. Brot., sér. 2, 41: 313 (1968) | Barbosa & Lemos 8381 | Gaza, Chibuto, Near Chibuto, road to Alto Changane | -24.6574 | 33.5652 | 1959 |  | Paratype (LISC) |
|  |  |  |  |  | Bol. Soc. Brot., sér. 2, 41: 313 (1968) | Exell & Mendonca 554 | Maputo, Goba | -26.2025 | 32.1321 | 1955 |  | Paratype (LISC) |
|  |  |  |  |  | Bol. Soc. Brot., sér. 2, 41: 313 (1968) | Exell & Mendonca 585 | Inhambane, Panda | -24.0637 | 34.7283 | 1955 |  | Paratype (LISC) |
|  |  |  |  |  | Bol. Soc. Brot., sér. 2, 41: 313 (1968) | Garcia 332 | Manica, Chimoio, Eastern slope of Garuzo Mountain | -18.9538 | 33.1663 | 1948 |  | Paratype (LISC) |
|  |  |  |  |  | Bol. Soc. Brot., sér. 2, 41: 313 (1968) | Garcia 262 | Manica, Chimoio, Nhamissanguere Forest, near the road to Gondola | -19.1051 | 33.5655 | 1948 |  | Paratype (LISC) |
|  |  |  |  |  | Bol. Soc. Brot., sér. 2, 41: 313 (1968) | Junod 230 | Maputo, Marracuene, Ricatta | -25.7660 | 32.6397 | 1917 |  | Paratype (LISC) |
|  |  |  |  |  | Bol. Soc. Brot., sér. 2, 41: 313 (1968) | Mendonca 3735 | Manica, Chimoio, Near Braunstein sawmill, Amatongas | -19.1834 | 33.7690 | 1948 |  | Paratype (LISC) |
|  |  |  |  |  | Bol. Soc. Brot., sér. 2, 41: 313 (1968) | Pimenta 17304 | Maputo, surroundings of Maputo | -25.8206 | 32.6579 | 1946 |  | Paratype (LISC) |
|  |  |  |  |  | Bol. Soc. Brot., sér. 2, 41: 313 (1968) | Quintas 74 | Maputo, Pessene | -25.6898 | 32.3872 | 1893 |  | Paratype (LISC) |
|  |  |  |  |  | Bol. Soc. Brot., sér. 2, 41: 313 (1968) | Salbany 88 | Manica, Moribane | -19.6988 | 33.4415 | 1942 |  | Paratype (LISC) |
|  |  |  |  |  | Bol. Soc. Brot., sér. 2, 41: 313 (1968) | Schlechter 11580 | Maputo | -25.4486 | 32.3984 | 1897 |  | Isoparatype (GRA) |
|  |  |  |  |  | Bol. Soc. Brot., sér. 2, 41: 313 (1968) | Simao 427 | Manica, Maronga savannah | -19.7511 | 33.2726 | 1945 |  | Paratype (LISC) |
|  |  |  |  |  | Bol. Soc. Brot., sér. 2, 41: 313 (1968) | Torre 7499 | Gaza, Limpopo, Around Guijá | -24.4353 | 32.9407 | 1948 |  | Paratype (LISC) |
|  |  |  |  |  | Bol. Soc. Brot., sér. 2, 41: 313 (1968) | Torre 2413 | Maputo, between Bilene and Maputo | -25.3456 | 32.8261 | 1940 |  | Paratype (LISC) |
|  |  |  |  |  | Bol. Soc. Brot., sér. 2, 41: 313 (1968) | Torre 7729 | Maputo, surroudings Bela Vista | -26.3419 | 32.6618 | 1948 |  | Paratype (WAG) |
|  |  |  |  |  | Bol. Soc. Brot., sér. 2, 41: 313 (1968) | Torre 7113 | Maputo, Montes de Goba | -26.2083 | 32.1416 | 1948 |  | Paratype (LISC) |
|  |  |  |  |  | Bol. Soc. Brot., sér. 2, 41: 313 (1968) | Torre 7499 | Gaza, Guijá, Limpopo, Guijá surroundings | -24.4335 | 32.9418 | 1948 |  | Paratype (LMU) |
| ***Tephrosia miranda* Brummitt** | Strict-Endemic | Shrub or subshrub | DD | Mozambique | Bol. Soc. Brot., sér. 2, 41: 387 (1968) | Torre & Paiva 11599 | Nampula, serra da Mesa, a ca. 6 km de Nampula | -15.1197 | 39.2647 | 1964 |  | Holotype (LISC) |
|  |  |  |  |  | Bol. Soc. Brot., sér. 2, 41: 387 (1968) | Torre & Paiva 9929 | Nampula, Monte Nassapo, 23 km from Nampula to Meconta | -15.1246 | 39.3958 | 1964 |  | Paratype (LISC) |
| ***Tephrosia montana* Brummitt** | Near-Endemic | Shrub or subshrub | NE | Zimbabwe to Mozambique | Bol. Soc. Brot., sér. 2, 41: 361 (1968) | Simao 1107 | Sofala, Gorongosa, Serra de Nhandete | -18.4713 | 34.1844 | 1946 |  | Paratype (LISC) |
|  |  |  |  |  | Bol. Soc. Brot., sér. 2, 41: 361 (1968) | Torre 6229 | Manica, Macequece | -18.9429 | 32.8668 | 1943 |  | Holotype (LISC) |
| ***Tephrosia paradoxa* Brummitt** | Native | Annual or biennial herb | LC | Tanzania to S. Tropical Africa | Bol. Soc. Brot., sér. 2, 41: 303 (1968) | Torre & Paiva 10687 | Niassa, Marrupa, 25 km from Marrupa to Máua | -13.3775 | 37.4114 | 1964 |  | Paratype (LISC) |
|  |  |  |  |  | Bol. Soc. Brot., sér. 2, 41: 303 (1968) | Torre & Paiva 10346 | Nampula, Ribáuè, 16 km from Ribabuè to Nampula | -15.0531 | 38.3627 | 1964 |  | Paratype (LISC) |
|  |  |  |  |  | Bol. Soc. Brot., sér. 2, 41: 303 (1968) | Torre & Paiva 10663 | Niassa, Marrupa, 36 km from Maúa to Marrupa | -13.7124 | 37.3365 | 1964 |  | Paratype (LISC) |
| *Tephrosia petersiana* Klotzsch [=*Mundulea sericea* (Willd.) A.Chev. subsp. sericea] |  |  |  |  | W.C.H.Peters, Naturw. Reise Mossambique 6(Bot., 2): 584 (1864) | Peters s.n. | Mozambique |  |  | 1843 |  | Isotype (K) |
| ***Tephrosia praecana* Brummitt** | Near-Endemic | Shrub or subshrub | VU | Zimbabwe to Mozambique | Bol. Soc. Brot., sér. 2, 41: 363 (1968) | Mendonca 2634 | Sofala: Manica, Mavita, Monte Chiroso | -19.5048 | 33.1394 | 1964 |  | Paratype (LISC) |
| ***Tephrosia purpurea* (L.) Pers. subsp. *altissima* Brummitt** | Native | Annual or biennial herb | NE | S. Tropical Africa | Bol. Soc. Brot., sér. 2, 41: 250 (1968) | Barbosa 1137 | Manica, Chimoio, Serra do Garuzo | -18.9632 | 33.1378 | 1948 |  | Holotype (LISC) |
|  |  |  |  |  | Bol. Soc. Brot., sér. 2, 41: 250 (1968) | Barbosa 1111 | Manica, Chimoio, Encosta da Serra do Garuzo | -18.9656 | 33.1569 | 1948 |  | Paratype (LISC) |
|  |  |  |  |  | Bol. Soc. Brot., sér. 2, 41: 250 (1968) | Barbosa 1152 | Manica, Chimoio, Bandula region | -19.0298 | 33.1553 | 1948 |  | Paratype (LISC) |
|  |  |  |  |  | Bol. Soc. Brot., sér. 2, 41: 250 (1968) | Barbosa 2439 | Niassa, between Fernao Veloso and Itocolo | -14.7098 | 40.5523 | 1948 |  | Paratype (LISC) |
|  |  |  |  |  | Bol. Soc. Brot., sér. 2, 41: 250 (1968) | Barbosa 1220 | Manica, Chimoio, Garuzo region | -18.9396 | 33.0656 | 1948 |  | Paratype (LISC) |
|  |  |  |  |  | Bol. Soc. Brot., sér. 2, 41: 250 (1968) | Barbosa 1137 | Manica, Chimoio, Serra do Garuzo | -18.9450 | 33.1511 | 1948 |  | Holotype (LISC) |
|  |  |  |  |  | Bol. Soc. Brot., sér. 2, 41: 250 (1968) | Barbosa 1200 | Manica, Chimoio, Serra do Garuzo | -18.9828 | 33.1681 | 1948 |  | Paratype (LISC) |
|  |  |  |  |  | Bol. Soc. Brot., sér. 2, 41: 250 (1968) | Barbosa 1152 | Manica, Chimoio, Bandula | -19.0117 | 33.1433 | 1948 |  | Paratype (LISC) |
|  |  |  |  |  | Bol. Soc. Brot., sér. 2, 41: 250 (1968) | Barbosa 1111 | Manica, Chimoio, Encosta da Serra do Garuzo | -18.9450 | 33.1511 | 1948 |  | Paratype (LISC) |
|  |  |  |  |  | Bol. Soc. Brot., sér. 2, 41: 250 (1968) | Barbosa 2439 | Nampula, Nacala, between Fernão Veloso and Itoculo | -14.5704 | 40.5992 | 1948 |  | Paratype (LISC) |
|  |  |  |  |  | Bol. Soc. Brot., sér. 2, 41: 250 (1968) | Garcia 539 | Manica, Chimoio, Serra do Garuzo | -18.9572 | 33.1608 | 1948 |  | Paratype (LISC) |
| ***Tephrosia purpurea* (L.) Pers. subsp. *dunensis* Brummitt** | Native | Perennial herb | NE | Africa to Arabian Peninsula, Tropical & Subtropical Asia to S. Pacific | Bol. Soc. Brot., sér. 2, 41: 251 (1968) | Torre & Paiva 11452 | Niassa, Mogincual, Quinga beach | -15.8475 | 40.2411 | 1964 |  | Paratype (LISC) |
| ***Tephrosia purpurea* (L.) Pers. subsp. *leptostachya* (DC.) Brummitt** | Native | Annual or biennial herb | NE | Africa, Sinai to Arabian Peninsula, Pakistan to India | Bol. Soc. Brot., sér. 2, 41: 245 (1968) | Torre 7828 | Gaza, Limpopo, Caniçado, between Dinga and Saúte. | -24.5140 | 33.0274 | 1948 |  | Paratype (LMU) |
| ***Tephrosia whyteana* Baker f. subsp. *gemina* Brummitt** | Strict-Endemic | Shrub or subshrub | CR | Mozambique | Bol. Soc. Brot., sér. 2, 41: 361 (1968) | Mendonca 2163 | Zambézia, Montes do Gúruè | -15.4481 | 36.9972 | 1944 | X | Holotype (LISC) |
| *Trachylobium mossambicense* Klotzsch [=*Hymenaea verrucosa* Gaertn.] |  |  |  |  | W.C.H.Peters, Naturw. Reise Mossambique 6(Bot., 1): 21 (1861) | Peters s.n. | Cabo Delgado, Quisanga | -12.4310 | 40.4934 | Unknown |  | Isotype (K) |
| *Vigna coerulea* Baker [=*Vigna unguiculata* (L.) Walp. subsp. *tenuis* (E.Mey.) Maréchal, Mascherpa & Stainier] |  |  |  |  | D.Oliver & auct. suc. (eds.), Fl. Trop. Afr. 2: 203 (1871) | Kirk 15 | Sofala, Luabo mouth of Zambesi | -18.4111 | 36.0693 | 1858 |  | Holotype (K) |
| ***Vigna junodii* Harms [=*Dolichos junodii* (Harms) Verdc.]** | Native | Perennial herb | NE | Mozambique to S. Africa | J. Linn. Soc., Bot. 30: 93 (1894) | Junod 208 | Mozambique |  |  | 1890 |  | Isosyntype (BR) |
|  |  |  |  |  | J. Linn. Soc., Bot. 30: 93 (1894) | Junod 209 | Mozambique |  |  | 1891 |  | Syntype (HBG) |
| ***Xylia mendoncae* Torre** | Strict-Endemic | Tree | VU | Mozambique | Contr. Conhecimento Fl. Mocambique 2: 94 (1954) | Mendonca 1913 | Inhambane, Vilanculos, near Mucoque | -21.9836 | 35.3172 | 1944 |  | Holotype (LISC) |
| ***Xylia torreana* Brenan** | Native | Tree | LC | S. Tropical Africa to NE. Limpopo | Kew Bull. 12: 359 (1957 publ. 1958) | Chase 2244 | Sofala, Maringua's village, 10 km N of River Save | -17.8895 | 34.3652 | 1958 |  | Holotype (K) |
|  |  |  |  |  | Kew Bull. 12: 359 (1957 publ. 1958) | Torre 6129 | Manica, between Espungabera e Chibabava | -20.3604 | 33.1542 | 1943 |  | Paratype (LISC) |
|  |  |  |  |  | Kew Bull. 12: 359 (1957 publ. 1958) | Torre 2721 | Inhambane, between Vilanculos and Funhalouro | -22.4026 | 34.6615 | 1941 |  | Paratype (LISC) |
| ***Zygia petersiana* Bolle [=*Albizia petersiana* (Bolle) Oliv.]** | Native | Tree | LC | S. Somalia to S. Tropical Africa | W.C.H.Peters, Naturw. Reise Mossambique 6(Bot., 1): t. 1 (1861) | Peters s.n. | Sofala, Boror and Sena | -17.4469 | 35.0359 | 1846 |  | Isotype (BM) |

^1^Taxa in bold represent those currently accepted. Growth form, conservation status (IUCN), native status, and global native distribution refer only to the 126 currently accepted taxa.

^2^Native status in Mozambique [sensu Darbyshire et al. (2019)].

^3^Conservation status according to IUCN Red List of Threatened Species (IUCN, 2021): CR, Critical endangered; EN, Endangered; VU, Vulnerable; NT, Near threatened; LC, Least concern; DD, Data deficient; NE, Not evaluated.

^4^The species is classified as Least Concern, but the subspecies is not evaluated.

*Specimens formerly held at the Herbarium of the Botanic Garden and Botanical Museum Berlin-Dahlem (B) and destroyed during the Second World War.

**Table S2.** Details on the Fabaceae type specimens’ collectors in Mozambique.

| **Collector Name** | **Collector Abbreviation** | **No. of Collected Specimens** | **No. of Locations (Provinces)** | **Minimum Year** | **Maximum Year** | **Collecting Activity (Years)** | **No. of Missing Dates** | **No. of Missing Locations** | **Index of Collector Importance** |
| --- | --- | --- | --- | --- | --- | --- | --- | --- | --- |
| António Rocha da Torre | Torre | 54 | 10 | 1934 | 1968 | 34 | 0 | 0 | 1.000 |
| John Kirk | Kirk | 29 | 6 | 1856 | 1862 | 6 | 0 | 0 | 0.479 |
| Wilhelm Peters | Peters | 26 | 7 | 1843 | 1846 | 3 | 0 | 3 | 0.463 |
| Luis Augusto Grandvaux Barbosa | Barbosa | 25 | 7 | 1947 | 1959 | 12 | 0 | 0 | 0.507 |
| Jorge Paiva | Paiva | 16 | 5 | 1963 | 1968 | 5 | 0 | 0 | 0.321 |
| Friedrich Schlechter | Schlechter | 14 | 2 | 1895 | 1898 | 3 | 0 | 0 | 0.200 |
| Thomas Sim | Sim | 10 | 3 | 1908 | 1908 | 0 | 0 | 2 | 0.175 |
| Francisco de Ascenção Mendonça | Mendonça | 9 | 5 | 1944 | 1964 | 20 | 0 | 0 | 0.343 |
| Manuel Fernandes Correia | Correia | 8 | 3 | 1948 | 1968 | 20 | 0 | 0 | 0.274 |
| António Gomes e Sousa | Gomes E Sousa | 8 | 4 | 1931 | 1947 | 16 | 0 | 0 | 0.280 |
| John Forbes | Forbes | 7 | 2 | 1822 | 1822 | 0 | 0 | 0 | 0.117 |
| Américo Pires de Lima | Pires De Lima | 7 | 2 | 1916 | 1917 | 1 | 0 | 0 | 0.122 |
| Aurélio Antero Balsinhas | Balsinhas | 6 | 2 | 1960 | 1961 | 1 | 0 | 0 | 0.113 |
| Francisco Leal de Lemos | Lemos | 6 | 3 | 1957 | 1961 | 4 | 0 | 0 | 0.161 |
| Helen G. Faulkner | Faulkner | 5 | 1 | 1946 | 1949 | 3 | 0 | 0 | 0.085 |
| Ladislau Menyhart | Menyhart | 5 | 2 | 1891 | 1902 | 11 | 0 | 0 | 0.162 |
| Eduardo Campos de Andrada | Andrada | 4 | 2 | 1948 | 1949 | 1 | 0 | 0 | 0.094 |
| M. Fidalgo de Carvalho | Carvalho | 4 | 2 | 1949 | 1949 | 0 | 0 | 0 | 0.088 |
| José Gonçalves Garcia | Garcia | 4 | 1 | 1948 | 1948 | 0 | 0 | 0 | 0.058 |
| Henri-Alexandre Junod | Junod | 4 | 1 | 1890 | 1917 | 27 | 0 | 1 | 0.217 |
| Carl Ernst Otto Kuntze | Kuntze | 4 | 0 | 1894 | 1894 | 0 | 0 | 4 | 0.028 |
| Joaquim Simão | Simão | 4 | 2 | 1945 | 1946 | 1 | 0 | 0 | 0.094 |
| Augusto De Carvalho | Augusto De Carvalho | 3 | 2 | 1884 | 1884 | 0 | 0 | 1 | 0.079 |
| Mary Agnes Chase | Chase | 3 | 2 | 1950 | 1958 | 8 | 0 | 0 | 0.126 |
| Arthur Wallis Exell | Exell | 3 | 2 | 1955 | 1955 | 0 | 0 | 0 | 0.079 |
| L. Marrime | Marrime | 3 | 1 | 1961 | 1961 | 0 | 0 | 0 | 0.049 |
| Hiram Wild | Wild | 3 | 2 | 1949 | 1955 | 6 | 0 | 0 | 0.114 |
| Charles Ernest Frank Allen | Allen | 2 | 1 | 1911 | 1911 | 0 | 0 | 0 | 0.039 |
| John Burrows | Burrows | 2 | 1 | 2006 | 2006 | 0 | 0 | 0 | 0.039 |
| Sandra Burrows | Burrows | 2 | 1 | 2006 | 2006 | 0 | 0 | 0 | 0.039 |
| R. Cecil Wood | Cecil | 2 | 1 | 1899 | 1899 | 0 | 0 | 0 | 0.039 |
| Luís Macuácua | Macuácua | 2 | 1 | 1960 | 1960 | 0 | 0 | 0 | 0.039 |
| B. M. Patel | Patel | 2 | 1 | 2007 | 2007 | 0 | 0 | 0 | 0.039 |
| A. Peter | Peter | 2 | 1 | 1925 | 1925 | 0 | 1 | 1 | 0.039 |
| Franz Stuhlmann | Stuhlmann | 2 | 1 | 1889 | 1889 | 0 | 0 | 1 | 0.039 |
| William Johnson | Johnson | 2 | 1 | 1907 | 1907 | 0 | 0 | 0 | 0.039 |
| T. Avis | Avis | 1 | 1 | 1998 | 1998 | 0 | 0 | 0 | 0.030 |
| Jeanne M. Borle | Borle | 1 | 1 | 1920 | 1920 | 0 | 0 | 0 | 0.030 |
| C. F. Johnson | Johnson | 1 | 1 | 1998 | 1998 | 0 | 0 | 0 | 0.030 |
| Frances Mary Chase (née Crawford) | Crawford | 1 | 1 | 2009 | 2009 | 0 | 0 | 0 | 0.030 |
| A. Esteves de Sousa | Esteves De Sousa | 1 | 1 | 1945 | 1945 | 0 | 0 | 0 | 0.030 |
| Carlo Antonio Fornasini | Fornasini | 1 | 1 | Unknown | Unknown | Unknown | 1 | 0 | Unknown |
| David Goyder | Goyder | 1 | 1 | 2009 | 2009 | 0 | 0 | 0 | 0.030 |
| Jan De Koning | Koning | 1 | 1 | 1981 | 1981 | 0 | 0 | 0 | 0.030 |
| Joseph Thomas Last | Last | 1 | 1 | 1887 | 1887 | 0 | 0 | 0 | 0.030 |
| Leslie C. Leach | Leach | 1 | 1 | 1962 | 1962 | 0 | 0 | 0 | 0.030 |
| João de Loureiro | Loureiro | 1 | 0 | 1770 | 1770 | 0 | 0 | 1 | 0.000 |
| Edouard Pierre Luja | Luja | 1 | 1 | 1901 | 1901 | 0 | 0 | 0 | 0.030 |
| Quentin Luke | Luke | 1 | 1 | 2009 | 2009 | 0 | 0 | 0 | 0.030 |
| José Aguiar Macedo | Macedo | 1 | 1 | 1974 | 1974 | 0 | 0 | 0 | 0.030 |
| Albert Oliver Dean Mogg | Mogg | 1 | 1 | 1962 | 1962 | 0 | 0 | 0 | 0.030 |
| J. J. Monteiro | Monteiro | 1 | 1 | 1876 | 1876 | 0 | 0 | 0 | 0.030 |
| Alfredo Nuvunga | Nuvunga | 1 | 1 | 1981 | 1981 | 0 | 0 | 0 | 0.030 |
| A. F. Patacas | Patacas | 1 | 1 | 1970 | 1970 | 0 | 0 | 0 | 0.030 |
| José Gomes Pedro | Pedro | 1 | 1 | 1948 | 1948 | 0 | 0 | 0 | 0.030 |
| José Pedrógão de Jesus | Pedrogão | 1 | 1 | 1948 | 1948 | 0 | 0 | 0 | 0.030 |
| A. Pimenta | Pimenta | 1 | 1 | 1946 | 1946 | 0 | 0 | 0 | 0.030 |
| Prelado | Prelado | 1 | 1 | 1894 | 1894 | 0 | 0 | 0 | 0.030 |
| Francisco Dias Quintas | Quintas | 1 | 1 | 1893 | 1893 | 0 | 0 | 0 | 0.030 |
| George Rogers | Rogers | 1 | 1 | 1915 | 1915 | 0 | 0 | 0 | 0.030 |
| A. Salbany | Salbany | 1 | 1 | 1942 | 1942 | 0 | 0 | 0 | 0.030 |
| Edmund André Charles Louis Eloi Schelpe | Schelpe | 1 | 1 | 1962 | 1962 | 0 | 0 | 0 | 0.030 |
| Ernst J. D. Schmidt | Schmidt | 1 | 1 | 2010 | 2010 | 0 | 0 | 0 | 0.030 |
| Kathy Sheppard | Sheppard | 1 | 1 | 1909 | 1909 | 0 | 0 | 0 | 0.030 |
| James Stewart | Stewart | 1 | 1 | 1862 | 1862 | 0 | 0 | 0 | 0.030 |
| Charles Swynnerton | Swynnerton | 1 | 1 | 1906 | 1906 | 0 | 0 | 0 | 0.030 |
| Jonathan Timberlake | Timberland | 1 | 1 | 2009 | 2009 | 0 | 0 | 0 | 0.030 |

**Table S3.** Summary statistics of generalised linear models (GLMs) comparing different combinations of predictor variables used to explain the spatial distribution of Fabaceae type specimen collections in Mozambique.

| **Model name** | **^a^AICc** | **^b^Pseudo-R^2^** | **Variables included** |
| --- | --- | --- | --- |
| All variables | 586.9191 | 0.549749 | Elevation, slope, LULC, distance to the nearest road and harbour |
| Environmental | 687.7045 | 0.4676031 | Elevation, slope, and LULC |
| Accessibility + LULC | 920.2012 | 0.2854445 | LULC, distance to the nearest road and harbour |
| Accessibility | 1145.269 | 0.1075587 | Distance to the nearest road and harbour |
| LULC | 1086.721 | 0.1518489 | LULC |
| Null | 1278.558 | NA | Intercept only |

^a^Akaike Information Criterion corrected for small sample sizes (AICc); ^b^McFadden's Pseudo-R² values.


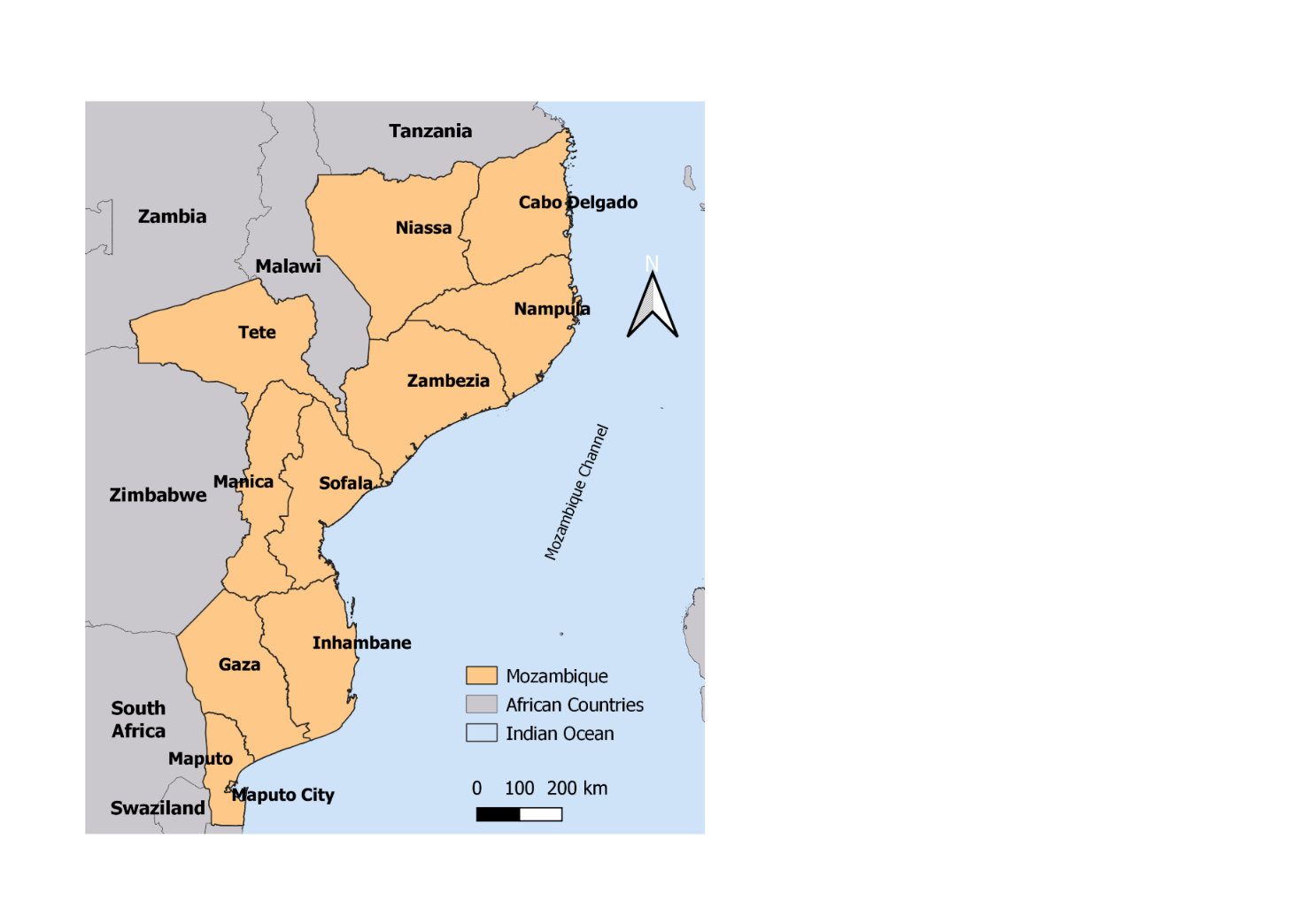


**Figure S1.** Map showing Mozambique as the study area with details on provinces, neighbouring African Countries and the Indian Ocean.


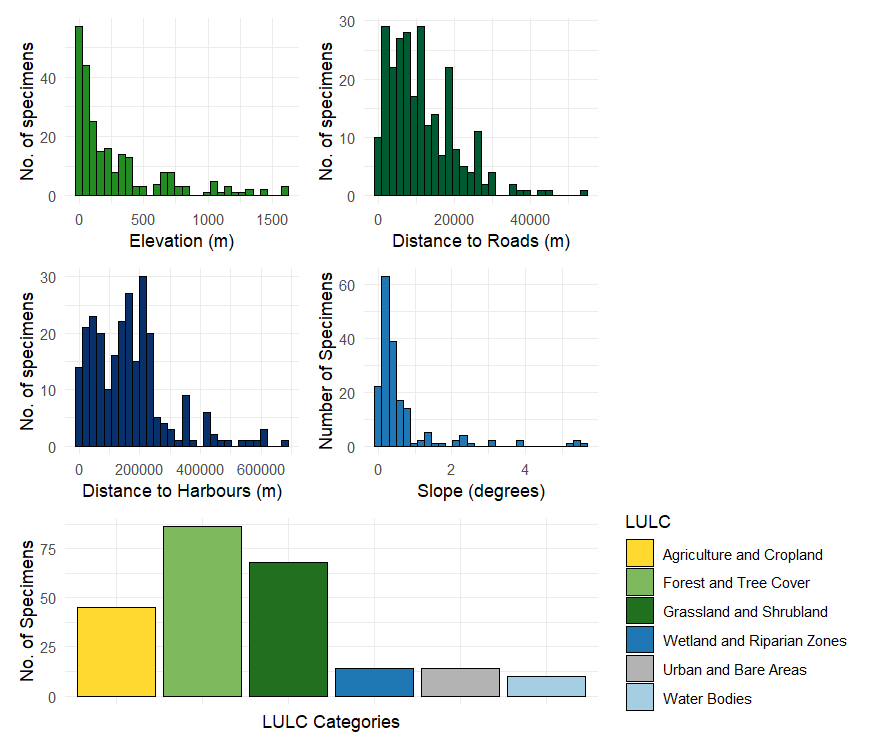


**Figure S2.** Exploratory analysis of predictor variables used in the generalised linear models (GLMs) of Fabaceae type specimen distribution in Mozambique. The plots show the distribution of environmental (elevation, slope, land cover) and accessibility (distance to the nearest road and harbour) variables across collection localities.


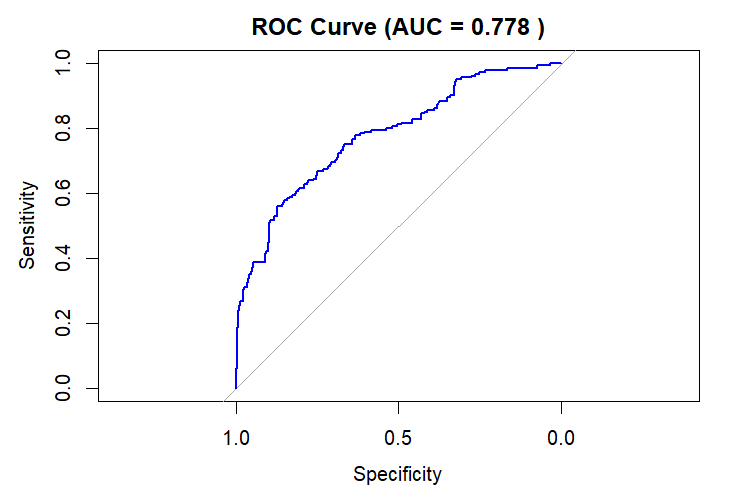


**Figure S3.** Receiver Operating Characteristic (ROC) curve for the generalised linear model (GLM) predicting the occurrence of Fabaceae type specimen collections in Mozambique. The Area Under the Curve (AUC) value indicates the model's discriminatory power, with values closer to 1 reflecting higher predictive accuracy.
